# Machine learning-driven identification of virulence determinants in *Borrelia burgdorferi* associated with human dissemination

**DOI:** 10.1371/journal.pcbi.1014407

**Published:** 2026-06-17

**Authors:** Hoa Thanh Nguyen, Catherine A. Brissette

**Affiliations:** Department of Biomedical Science, University of North Dakota School of Medicine and Health Science, Grand Forks, North Dakota, United States of America; Indian Institute of Technology Hyderabad, INDIA

## Abstract

Lyme disease, the most common tick-borne infectious disease in the United States, presents with highly variable clinical outcomes, ranging from localized erythema migrans to severe disseminated complications affecting the heart, joints, and nervous system. The bacterial determinants underlying this phenotypic variation remain largely unknown, limiting our ability to predict disease progression and optimize treatment strategies. Here, we applied machine learning (ML) approaches to identify specific amino acid residues within surface-exposed virulence factors that predict human dissemination phenotypes. Utilizing the published whole genome sequences from 299 clinical *Borrelia burgdorferi* isolates collected from the United States and Slovenia over a 30-year period (1992–2021), we extracted and characterized translated amino acid sequences (variants) of seven known virulence factors (BB_0406, BBK32, DbpA, OspA, OspC, P66, and RevA). Protein variants were classified based on their association with disseminated versus localized infections using clinical metadata. Cramér’s V analysis revealed possible strong associations between dissemination phenotypes and five adhesins: BBK32, DbpA, OspC, P66, and RevA. We developed ML models using five algorithms with multiple feature selection strategies, achieving robust predictive performance for DbpA, OspC, and RevA variants (all performance metrics > 0.7). Feature importance analysis identified 57, 29, and 42 key predictive residues for DbpA, OspC, and RevA, respectively. Notably, B-cell epitope prediction revealed significant enrichment of ML-identified residues within predicted epitope regions for OspC (11 overlapping residues, OR = 3.57, *p* = 0.006) and RevA (12 overlapping residues, OR = 2.37, *p* = 0.048), suggesting these residues may influence immune recognition and bacterial persistence. This study establishes the first computational framework linking Borrelia protein sequence variants to clinical dissemination phenotypes, providing molecular insights into Lyme disease pathogenesis that may inform the development of improved diagnostics and therapeutic targets.

## Author summary

Lyme disease, caused by *Borrelia burgdorferi* (Bb), afflicts over 470,000 Americans annually, yet predicting which infections will progress from localized to disseminated disease remains a critical clinical challenge. Despite decades of research, the bacterial factors underlying these divergent disease trajectories have remained poorly understood. To address this gap, we leveraged the largest collection of clinical Bb genomes to date and applied machine learning to identify specific amino acid residues within key membrane surface proteins that distinguish invasive from non-invasive strains. Our analysis reveals that many of these predictive residues overlap with immune recognition and host-protein binding sites, suggesting a mechanistic link between sequence variation, immune evasion, and disease severity. This computational framework provides a foundation for investigating key virulence factors in Bb pathogenesis and developing rapid, sequence-based diagnostic tools that could guide treatment strategies. Beyond Lyme disease, our methodology demonstrates broad applicability to other bacterial pathogens where strain-level variation influences clinical outcomes.

## 1. Introduction

Lyme disease (LD) is an emerging infectious tick-borne disease in the northern hemisphere, with over 470,000 cases diagnosed and treated annually in the United States [1]. The causative agents are spirochetes belonging to the *Borrelia burgdorferi sensu lato* complex, referred to as Lyme Borrelia (LB). In North America, *B. burgdorferi sensu stricto* (hereafter Bb) predominates. Clinical manifestations range from localized erythema migrans to severe disseminated complications including Lyme arthritis, carditis, and neuroborreliosis, with treatment outcomes and disease severity varying widely among patients [2,3]. Understanding the bacterial determinants that influence disease progression from localized to disseminated infection is crucial for improving diagnostic strategies, treatment protocols, and patient outcomes.

LB exhibits a remarkable level of genetic diversity [4,5]. Its genome consists of an approximately 900 kb linear chromosome and about 21 plasmids (9 circular and 12 linear) ranging from 5 to 84 kb in size [5–7]. The genomic background, particularly plasmid content, affects its ecological, epidemiological, and pathogenic properties [8–14]. Clinical and animal studies have demonstrated that variations in plasmid content and sequence diversity of virulence factors influence spirochete dissemination and disease severity in mice and humans [15–17]. However, the segmented genome and high plasmid variability create technical challenges for sequencing, assembly, and analysis of the LB genome, ultimately limiting the development of consistent diagnostics and treatments. Since the publication of the first complete Bb genome in 1997 [7], numerous efforts have been made to sequence and map LB genomes [4,8,18–20]. In 2023, Lemieux et al. published the first large-scale long-read whole genome sequence (WGS) of human Bb isolates [21], providing a valuable resource for examining associations between Bb genotypes and clinical outcomes.

Bb is a Gram-negative bacterium with an atypical cell envelope lacking lipopolysaccharide, but enriched with outer membrane glycolipids and lipoproteins that are highly immunoreactive [22,23]. Many of these lipoproteins function as virulence factors critical for host-pathogen interactions, including vascular transmigration, immune invasion, and tissue adhesion. By analyzing the WGS of 299 human isolates, Lemieux et al., (2023) demonstrated that surface-exposed lipoproteins encoded on a unique set of plasmids and several loci are linked to dissemination. Several studies have reported that these surface-exposed lipoproteins bind host extracellular matrix (ECM) components, including fibronectin [24–26], glycosaminoglycans (GAGs) [27–29], as well as the plasma protein plasminogen [30–33]. Despite the established importance of these surface lipoproteins in pathogenesis, the specific gene/protein sequence variations that determine their functional differences and contributions to dissemination capacity remain poorly characterized.

Machine learning (ML) for classification tasks has been used for decades in biomedical studies. The application of ML approaches to pathogen genomics has shown remarkable success in identifying novel virulence determinants, predicting antimicrobial resistance, and classifying disease severity across various bacterial pathogens [34–36]. In contrast to traditional Genome-wide association studies (GWAS) and univariate statistical methods, which typically examine genetic variants independently under linear or additive assumptions and struggle with multiple testing corrections and identifying epistatic interactions, ML approaches can simultaneously evaluate multiple features, making them adept at detecting subtle patterns in high-dimensional genomic data [37,38]. Algorithms such as Generalized Linear Model (GLM), Principal Component Analysis Neural Network (PCANN), Partial Least Square (PLS), Support Vector Machine (SVM), and Random Forest (RF) are well-suited for high-dimensional, small-sample-size datasets, feature selection, and overfitting control [39–44]. These tools have also proven their efficiency in supporting biological interpretation and biomarker discovery [45–49].

This study aimed to identify specific amino acid (aa) residues within well-characterized LB surface-exposed virulence factors that could serve as biomarkers for predicting human dissemination phenotypes. We integrated genomic data from a large collection of clinical isolates with advanced computational methods. The surface-exposed proteins examined (BB_0406, BBK32, DbpA, OspA, OspC, P66, and RevA) were selected based on their established roles in host cell adhesion, immune evasion, and tissue invasion processes critical to bacterial dissemination. Using multiple ML algorithms, feature selections, and resampling methods, we successfully developed highly accurate predictive models for DbpA, OspC, and RevA aa sequences. This computational framework demonstrates the potential of protein sequence-based virulence prediction and provides molecular insights that could advance LD research.

## 2. Materials and methods

### 2.1. Data collection

Raw whole genome sequence reads and associated metadata of 299 clinically isolated Bb strains were downloaded from NCBI BioProject PRJNA923804 [21]. These isolates were collected from 299 patients from the Northeastern US (n = 201), Midwestern US (n = 62), and Slovenia (n = 36) over a period of 30 years (1992–2001). Of the 299 isolates, 118 (39.5%) and 173 (57.9%) were classified as Disseminated or Localized by a positive blood or CSF culture or a positive PCR on a blood sample, the presence of multiple EM lesions, and/or signs of neurological involvement. 8 of the 299 has no clinical records.

DNA sequences were trimmed and assembled using Trimmomatic v0.39 [50] and SPAdes 3.15.5 [51]. Assembled sequences were annotated based on B31 reference sequences [7] using Prokka [52]. Translated aa sequences of BB_0406, BBK32, DbpA, OspA, OspC, P66, and RevA from all isolates were collected into a single fasta file per protein. Using R packages DECIPHER v2.28.0 [53] and Biostrings v2.68.1 [54], we identified a unique set of sequences for each protein. We defined each unique aa sequence as a protein variant, which is distinct from protein isoforms generated from alternative splicing or post-translational modifications. All the tested proteins are membrane proteins with signal sequences that are cleaved during translocation and do not contribute to mature protein function. Since signal sequences are not functionally relevant and variations within them can introduce misalignments, signal sequences were removed prior to sequence alignment using AlignSeqs() function in DECIPHER. To assign names to each sequence, we calculated the normalized Hamming distance between each sequence and the B31 reference sequence using DistanceMatrix() function in DECIPHER (method = ‘overlap’, includeTerminalGaps = TRUE, and correction = “none”). Normalized Hamming distance represents the proportion of aa differences between aligned sequences, with values ranging from 0 (identical sequences) to 1 (completely different sequences). The resulting distances were ranked in ascending order, and sequences were named based on their rank (e.g., BB_0406_1). For OspC, sequences were named as the OspC type information from the metadata file provided by Lemieux et al. [21]. We identified 33 unique sequences of OspC that mapped to 23 known OspC types (A, B, B3, C, D, E, E3, F, F3, G, H, I, J, K, L, M, N, O, Q, R, S, T, and U). When there were more than one unique sequence mapped to one OspC type, an additional number was added after OspC type to indicate variants in the OspC type (e.g., B_1 and B_2).

Information of all unique sequences of the seven proteins is listed in S1 Table.

### 2.2. Cramér’s V association analysis

To assess the strength of association between two categorical variables (protein variants and dissemination phenotype), Cramér’s V analysis was used [55]. Cramér’s V is the expansion of the chi-square test for contingency tables bigger than 2 × 2. Cramér’s V values range from 0 (no association) to 1 (perfect association). A value bigger than 0.25 is considered a strong relationship [56].

Contingency with any expected frequency < 1 or with more than 20% of cells having expected frequencies <5 (i.e., sparse cells) can bias statistical results [57]. To mitigate this, we collapsed variant categories when any cell had an expected frequency <1 or when both dissemination categories had expected frequencies < 5, ensuring that fewer than 20% of sparse cells were in all the contingency tables. Cramér’s V was computed for both the original and collapsed contingency tables using the assocstats() function in the vcd R package (v1.4.12) [58]. Statistical significance of associations was assessed using chi-square and Fisher’s exact tests with Monte Carlo-simulated p-values, as implemented in the stats R package (v4.3.2) [59].

To identify individual variants enriched or depleted across dissemination phenotypes, standardized residues (stdres) were extracted from chi-square analysis. Variants with |stdres| > 2 (*p* < 0.05) were considered significantly associated with a phenotype. Enrichment ratios (Observed/Expected) were calculated for each variant-phenotype combination, with values > 1 indicating enrichment and < 1 indicating depletion.

### 2.3. Machine learning modeling of dissemination potential

#### 2.3.1. Data preprocessing.

For each protein, unique protein sequences were aligned, and signal sequences were removed. Each sequence was transformed to a binary feature matrix using one-hot encoding, a standard orthogonal encoding technique for aa sequences [60]. Each individual sequence matrix had dimensions L × D, where L represents the sequence length and D = 21 (20 standard aa plus 1 gap character “-”, which represents potential indels as determined by sequence alignment). The individual matrices were concatenated to form a single feature matrix of shape (N, L × D), where N is the total number of sequences. To reduce dimensionality, feature columns that were invariant across all sequences (i.e., positions with identical aa) were removed, as these provide no discriminative power. The feature (residue) names are formatted as X followed by a position number and an aa abbreviation. The position number represents the residue position in the consensus sequence derived from the alignment of all variants in each protein. A period after the position number indicates a gap position (e.g., X56.). To enable comparison with the correct residue position in the B31 sequence, we provide the B31 position and residue name in brackets after the ML residue name. For example, X46T indicates position 46 with a threonine residue in the ML models, and X46T(_B31_64A) shows that this corresponds to position 64 with an alanine in the B31 sequence. The resulting matrix was used as the input feature matrix for ML modeling.

Unique sequences were classified into two groups based on their dissemination potential for ML analysis. A sequence was assigned to the Dis (Disseminated) group if at least one isolate carrying that sequence was clinically classified as Disseminated. A sequence was assigned to the Non-dis (Non-disseminated) group if all isolates carrying that sequence were clinically classified as Localized. If clinical status was unavailable for all isolates associated with a sequence, the sequence was assigned a NA label and removed from further analysis.

#### 2.3.2. Feature selection.

Feature selection is a widely used strategy in ML to select a subset of features from the original set, aiming to optimize learning performance while reducing computational expense [61,62]. In this study, we employed both supervised and unsupervised feature selection approaches to generate seven different feature sets for modeling each protein.

**Supervised feature selection:** Features were ranked using model-independent Variable Importance (VIP) scores calculated as the Receiver Operating Characteristic Area Under the Curve (ROC-AUC) for each individual feature. A VIP score of 0.5 indicates no discriminative power. Four subsets of features were created: (1) fs055 - features with VIP ≥ 0.55, and (32–4) ImpQ1, ImpQ2, and ImpQ3 - features with VIP scores above the first, second, and third quartiles, respectively.

**Unsupervised feature selection**: Features were selected based on variance filtering. Three feature sets (VarQ1, VarQ2, and VarQ3) were created using the first, second, and third quartiles of features’ variance as selection thresholds. The number of features in the original data and each subset are listed in S2 Table.

#### 2.3.3. Model training and cross-validation.

The dataset presented two main challenges: small sample size and class imbalance, with the Dis class containing about twice the number of sequences in the Non-dis class. To address class imbalance, we applied Synthetic Minority Oversampling Technique (SMOTE) to oversample the minority Non-dis class [63].

We employed a nested cross-validation strategy to provide robust and unbiased model evaluation. Specifically, the outer loop generated 100 unique train-test partitions (25 repetitions x 4-fold CV, 75% train/25% test). This multi-split approach provided 100 independent estimates of model generalization performance, reducing dependency on any single data split. The inner loop optimized hyperparameters using two complementary resampling techniques: Leave-One-Out Cross Validation (LOOCV) and bootstrap (n = 100 iterations), exclusively within training folds to prevent data leakage and provide unbiased assessment on unseen data.

Five classification algorithms were chosen for their diverse model paradigms and complementary strengths on handling high-dimensional data: Partial Least Square Discriminant Analysis (PLS), Random Forest (RF), Support Vector Machine with a radial basis function kernel (SVM-RBF), elastic-net regularized Generalized Linear Model (GLMnet), and Principal Component Analysis Neuron Network (PCANN). PLS reduced dimensionality while maximizing covariance between features and class labels. RF and SVM-RBF captured non-linear relationships, with RF modeling feature interactions through ensemble decision trees and SVM-RBF learning maximal-margin boundaries in a kernel-transformed space. GLMnet performed sparse feature selection and mitigated overfitting through combined L1/L2 regularization, while PCANN integrated PCA-based dimensionality reduction with a neural network to model complex non-linear relationships. Together, these algorithms allowed us to capture patterns ranging from linear to highly non-linear while balancing predictive performance and interpretability.

**Fig 1:**
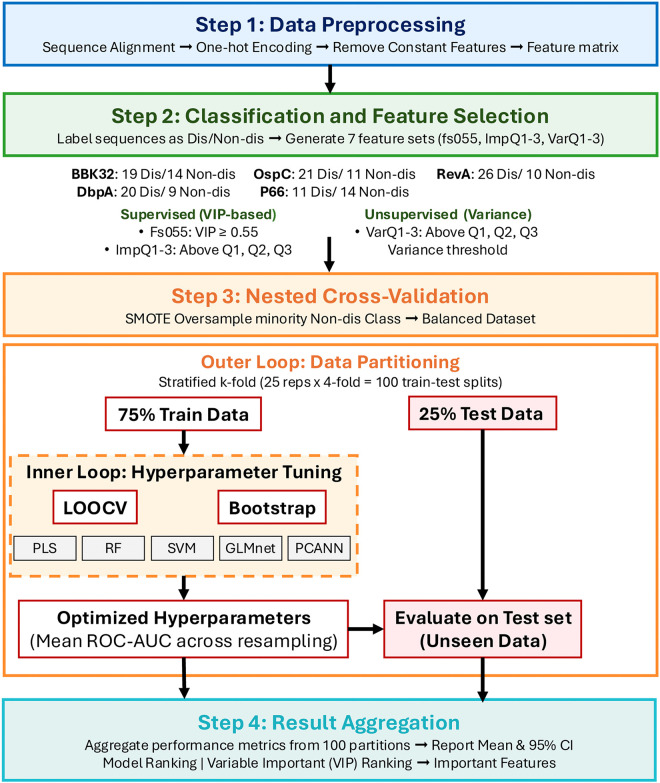
Overview of machine learning workflow.

For each algorithm and training fold, hyperparameters were tuned using a grid search with 15 tuning length steps, optimizing for ROC-AUC. Parallel processing across 36 cores was implemented to enhance computational efficiency. The overall ML workflow is summarized in Fig 1. In total, 70 models were generated per protein (2 resampling methods × 5 algorithms × 7 feature sets). All analyses were performed in R (v 4.3.2) using the packages pls, randomForest, e1071, glmnet, and nnet, implemented through the caret (v6.0) framework for unified model training, tuning, and evaluation [59,64].

#### 2.3.4. Model performance evaluation.

The performance of models was evaluated using multiple metrics: ROC-AUC, accuracy, sensitivity (true positive rate, TPR), and specificity (true negative rate, TNR). Class predictions for accuracy, sensitivity, and specificity were derived from predicted probabilities using a decision threshold of 0.5. Sensitivity represents the proportion of actual positive (Dis) cases correctly identified, while specificity represents the proportion of actual negative (Non-dis) cases correctly identified (equivalent to 1 – False Positive Rate (FPR)). The ROC curve was generated by plotting the TPR against the FPR across all possible classification thresholds, with the ROC-AUC providing a threshold-independent measure of model discriminative ability. Values closer to 1.0 indicate better discrimination between classes, while 0.5 represents random classification.

For each model configuration (algorithm × feature set × resampling method), performance metrics were calculated independently for each of the 100 train-test splits and summarized as mean ± SD, together with 95% CI. Both training and test set performances were recorded to enable bias assessment.

To select the best model for each protein, we applied a composite score approach that equally weighted three key performance metrics (ROC-AUC, sensitivity, and specificity):


$$\begin{array}{c} \text{Composite Score}=(\text{ROC}-\text{AUC}+\text{Sensitivity}+\text{Specificity})\;/\;\text{3} \end{array}$$

ROC-AUC was used as the tiebreaker when multiple models achieved identical Composite Score. This approach ensures selection of models with balanced performance across both positive and negative classes while maintaining strong overall discriminative ability. For each protein, the best model was independently selected for each resampling strategy (LOOCV and Bootstrap).

#### 2.3.5. Statistical comparison of resampling methods.

Performance comparisons between two resampling methods were conducted using paired t-tests across all model configurations. Model stability was quantified using coefficient of variation (CV), with lower values indicating greater reproducibility. Generalization gap, defined as the difference between train and test performance (Bias = Train – Test), was quantified to assess overfitting (positive values) or underfitting (negative values) with comparison conducted across all stratification levels.

Agreement between LOOCV and bootstrap methods was assessed using Pearson correlation analysis and Bland-Altman plots. Correlation coefficients (r) and associated *p*-values were calculated for each performance metric across all models from five proteins. Bland-Altman analysis quantified systematic bias (mean difference) and random variation (95% limits of agreement, LOA) between the two resampling methods.

#### 2.3.6. Feature importance analysis.

VIP scores, which represent an overall measure of feature contribution to the model’s predictive performance, were computed for each model using the varImp() function implemented in the caret R package [64]. For GLMnet, VIP was derived from the absolute values of the coefficients at the optimal regularization parameter. For PLS, VIP was based on weighted sums of absolute regression coefficients across latent components, where weights are proportional to the reduction sums of squares. For RF, VIP was computed from the mean decrease in Gini impurity across all trees in the ensemble. For SVM-RBF and PCANN, VIP was estimated using a model-agnostic sensitivity measure that quantifies the influence of each predictor on the model output. In all cases, VIPs were scaled to a 0–100 range to facilitate comparability across models.

Feature importance was aggregated across all 100 outer train-test partitions using a stability-selection-inspired procedure [65]. For each model and each train-test partition, features were ranked by their VIP scores, and the top 20 features were retained. Feature importance was then summarized as the frequency with which each feature appeared among the top-ranked features across all 100 partitions. Features were considered important if this frequency exceeded 40 out of 100 partitions. This frequency-based aggregation provided a stability-aware measure of feature importance, reducing sensitivity to individual train-test partitions.

The final set of important features for each protein was determined by taking the union of: (1) the intersection of important features identified consistently across all supervised feature selection models (fs055, ImpQ1, ImpQ2, ImpQ3), and (2) the intersection of important features identified consistently across all unsupervised feature selection models (VarQ1, VarQ2, VarQ3). This conservative selection strategy ensured that only features with robust and reproducible important signals were retained for downstream interpretation.

### 2.4. B-cell epitope prediction

To predict conformational B-cell epitopes in Bb proteins, we employed the DiscoTope 3.0, a robust structure-based epitope prediction tool that integrates inverse folding latent representations and a positive-unlabeled learning strategy [66]. DiscoTope 3.0 has been benchmarked to maintain high predictive performance across solved, relaxed, and predicted structures and outperformed other prediction tools in predicted structures.

3D protein structures of DbpA, OspC, and RevA were generated using the AlphaFold 3.0 server [67] based on the protein sequences in the B31 strain. The protein structures were submitted to the DiscoTope 3.0 server (https://services.healthtech.dtu.dk/services/DiscoTope-3.0/), which was run using the default parameter (Epitope confidence threshold (calibrated scores) at moderate confidence).

DiscoTope 3.0 computes an epitope propensity score for each surface-exposed residue, based on updated statistics from a larger set of antigen-antibody complex structures [66]. Residues with a calibrated score above the default threshold for moderate confidence (0.9, recall up to ~50% for moderate confidence) were classified as part of a predicted conformational B cell epitope. All predicted residues were visualized and mapped onto the protein structure using Chimera X [68] for spatial localization and further interpretation.

### 2.5. Host-pathogen protein-protein interaction (PPI) site prediction

We computed protein-protein complex structures of DbpA-decorin, OspC-OspC-plasminogen, and RevA-fibronectin using the AlphaFold 3 server. For DbpA, OspC, and RevA, the aa sequences in the B31 strain were used, and signal peptides were removed. Sequences of human decorin, plasminogen, and fibronectin were accessed from NCBI NP_001911.1, AAA60113.1, and NP_997647.2, respectively. The sequences of leucine-rich repeat regions in decorin (residues 52–359), five kringle domains in plasminogen (residues 99–570), and 70 kDa regions in fibronectin (residues 52–608), which are known interaction sites with DbpA [69], OspC [70], and RevA [24], respectively, were used to generate the protein-protein structures. The best predicted structures were submitted to Chimera X for visualization and spatial localization.

## 3. Results

### 3.1. Associations of protein variants and dissemination phenotype

We extracted gene sequences of 7 known Bb virulence factors from the published WGS of 299 clinical isolates (NCBI BioProject PRJNA923804). Following translation into aa sequences, we identified between 4 and 36 unique variants per protein. Pairwise aa sequence alignment with the B31 reference sequence revealed distinct patterns of diversity across 7 proteins (Table 1). DbpA, OspC, and RevA exhibited dispersed variations across multiple sites (mean identity 77.90%, 75.11%, and 73.78%, respectively), with the majority of the variants showing >10% substitution rates. In contrast, BB_0406, BBK32, OspA, and P66 showed concentrated variations at a few sites (mean identity 97.53-99.36%) with all the variants showing <5% substitution rates.

**Table 1:** Sequence identity ranges and substitution patterns of tested proteins.

Protein	#Variants^a^	Identity range^b^	Mean^c^	Pattern^d^	≤5% substitutions^e^	>10% substitutions^f^
**BB_0406**	4	98.92-99.46	99.28	Concentrated	3	0
**BBK32**	34	94.83-99.71	97.54	Concentrated	32	0
**DbpA**	30	65.14-98.29	77.90	Dispersed	1	28
**OspA**	13	98.43-99.61	98.96	Concentrated	12	0
**OspC**	33	70.5-81.5	75.11	Dispersed	0	32
**P66**	25	96.99-99.83	99.37	Concentrated	24	0
**RevA**	36	65.25-99.29	73.78	Dispersed	6	29

^a^Number of unique sequences, ^b,c^ Identity range and mean pairwise identity compared to the B31 sequence, (excluding 100% match). ^d^ Substitution patterns classified as “Concentrated” if ≤ 5% substitutions in most variants, or “Dispersed” if >10% substitutions in most variants. ^e,f^ Number of variants with ≤ 5% or > 10% substitutions, respectively.

**Fig 2:**
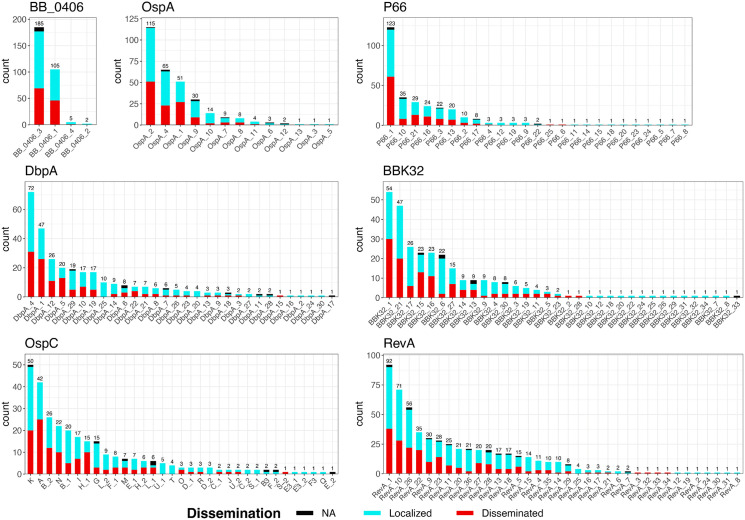
Profile of unique protein sequences of BB_0406, BBK32, DbpA, OspA, OspC, P66, and RevA. Each bar represents the number of isolates carrying the corresponding sequences, with the exact count shown at the top of each bar.

The distribution of the protein variants with respect to clinical dissemination status is presented in Fig 2. Most variants were observed in both disseminated and localized (non-disseminated) strains. A subset of variants appeared to be exclusively associated with one clinical outcome. It is critical to note that most of the variants with exclusive outcomes were represented by very small sample sizes with ≤ 3 isolates per variant (1% of total isolates), limiting the strength of inference. As such, these apparent exclusive associations could arise by chance due to sampling limitations or under-representation. Nevertheless, rare variants may still reflect true biological associations, particularly given that isolates were collected over a 30-year period and may represent distinct evolutionary lineages or ecological niches. Among variants exclusively associated with localized infections, several had slightly larger sample sizes that provide somewhat more robust support: DbpA_20 (4 cases), DbpA_25 (10 cases), OspC type T (4 cases), and OspC type U_1 (5 cases), and RevA_24 (4 cases). Despite these limitations, the overall pattern of variant distribution suggests that some protein variants may be preferentially associated with either localized or disseminated disease, supporting their potential relevance in virulence and host-pathogen interaction dynamics.

**Fig 3:**
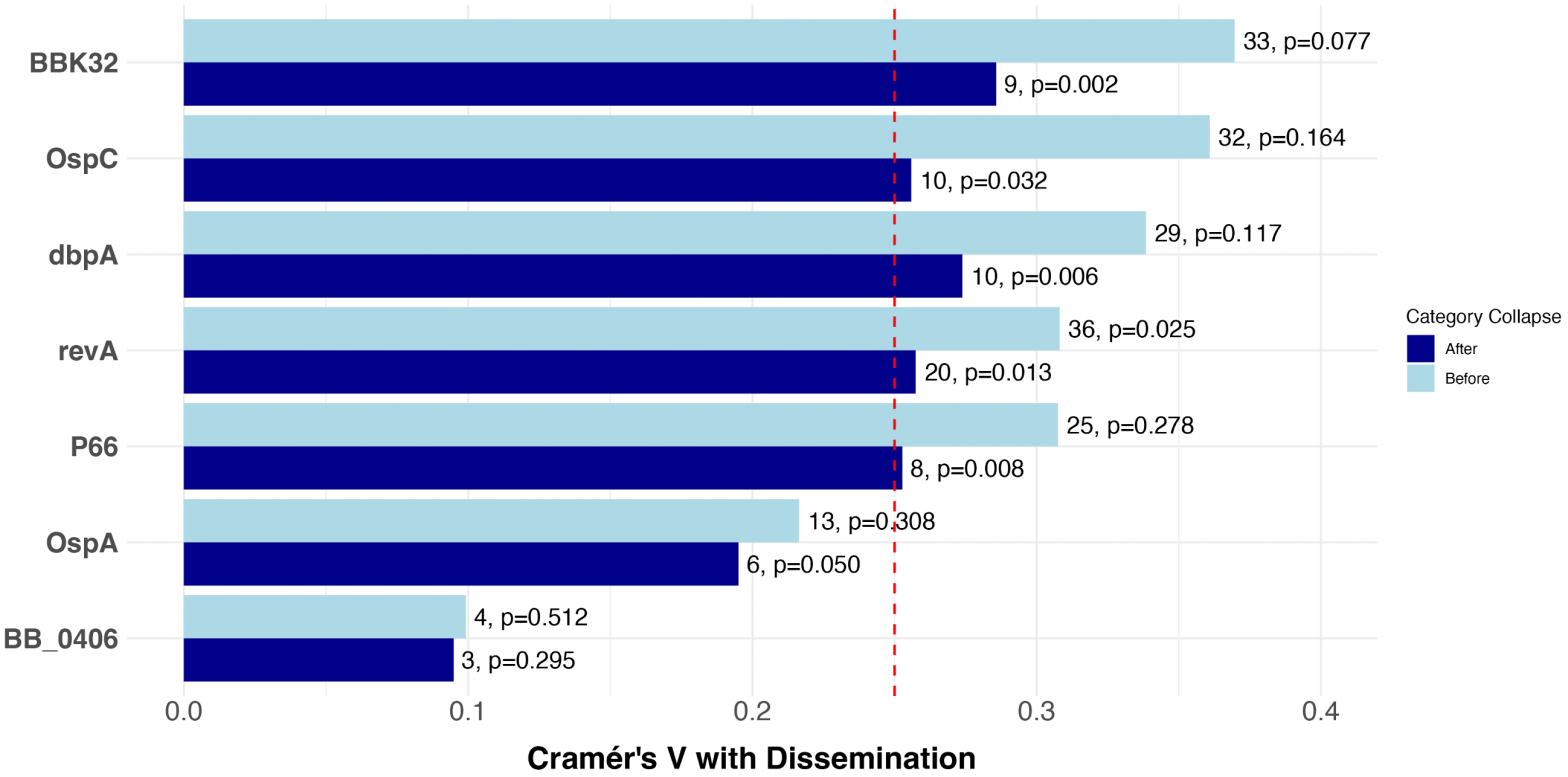
Association of protein variants with dissemination phenotype by Cramér’s V analysis. Bar length represents the strength of association before (light blue) and after (dark blue) grouping rare variants. Adjacent values indicate the number of protein variants and Fisher’s exact test *p*-value. A dashed red line at Cramér’s V = 0.25 marks the threshold for a strong association [56].

To assess the strength of association between protein variants and clinical dissemination status, we performed Cramér’s V analysis. Our analyses involved 2 × k contingency tables (k = 4–36 categories) with sample sizes ranging from n = 282–299, depending on the available data for each protein. To mitigate sparse contingency tables, we also calculated Cramer’s V after collapsing all the variant categories with any cell having an expected frequency < 1 or both dissemination categories having expected frequencies < 5 to one category. Following Akoglu’s guideline [56], we considered Cramér’s V > 0.25 as indicative of a strong association. Using this criterion, BBK32, DbpA, OspC, P66, and RevA showed strong associations with dissemination both before and after collapsing sparse categories (Fig 3). These five proteins were therefore selected for subsequent ML modeling to classify variants as associated with either disseminated or localized infections. In contrast, BB_0406 and OspA did not exhibit strong associations with clinical phenotypes, partly due to their low variant diversity (4 and 13 variants, respectively). Due to insufficient statistical signal, these two proteins were excluded from ML analysis.

In addition, we calculated the standard deviation (stdres) from the chi-square analysis of each variant-phenotype combination to identify specific variants enriched in disseminated and localized phenotypes. BBK32_1, DbpA_1, DbpA_5, OspC type A, OspC type H_1, P66_1, and RevA_22 were significantly enriched in disseminated phenotype, while BBK32_6, DbpA_25, OspA_10, and RevA_36 were significantly enriched in localized phenotype (enrichment ratio > 1, stdres > 2, *p* < 0.05, Table 2).

**Table 2:** Protein variant enrichment associated with dissemination phenotype.

Protein	Variant	Phenotype	Observed	Expected	Enrichment	stdres	p value
BBK32	BBK32_1	Disseminated	30	21.6	1.39	2.582	0.010
	BBK32_6	Localized	18	12.0	1.50	2.847	0.004
DbpA	DbpA_1	Disseminated	26	18.7	1.39	2.368	0.018
	DbpA_5	Disseminated	13	8.0	1.63	2.384	0.017
	DbpA_25	Localized	10	6.0	1.66	2.615	0.009
OspA	OspA_10	Localized	12	8.3	1.44	2.051	0.040
OspC	A	Disseminated	25	17.2	1.45	2.647	0.008
	H_1	Disseminated	10	6.1	1.63	2.078	0.038
P66	P66_1	Disseminated	61	48.3	1.26	3.087	0.002
RevA	RevA_22	Disseminated	20	12.5	1.59	2.722	0.006
	RevA_36	Localized	21	14.8	1.42	2.777	0.005

### 3.2. Prediction ability of trained models

**Fig 4:**
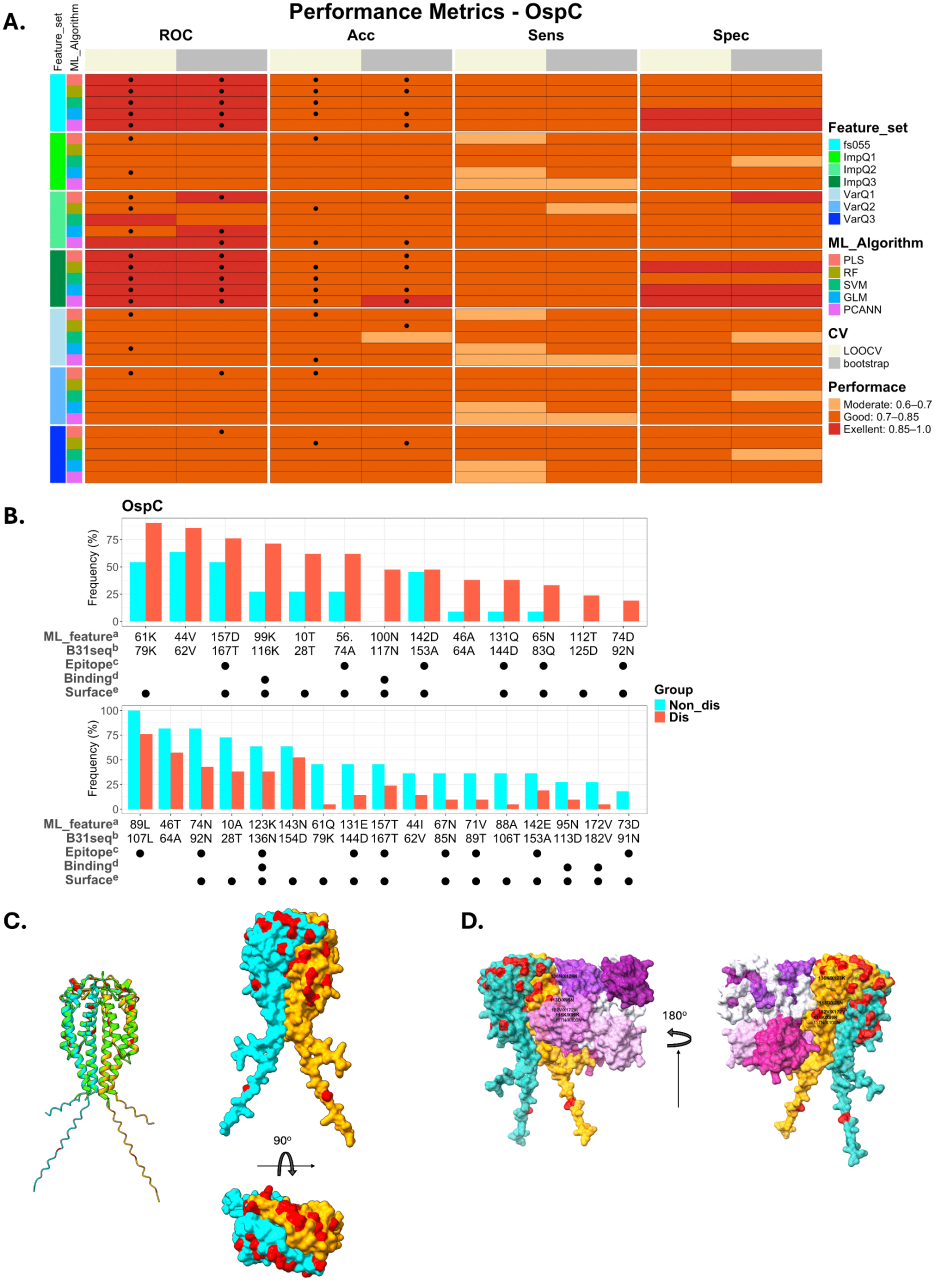
Machine learning model performance and predictive features in OspC. **(A)** Heatmap displaying average performance values for four key metrics (ROC, Acc, Sens, Spec) evaluated with LOOCV and Bootstrap on test datasets. Each cell represents the mean performance value across 100 random train-test splits. Black dots indicate statistically precise estimates (95% CI width < 0.05). Row annotations display the feature sets and ML algorithm as color-coded bar. See figure legend for color code. **(B)** Bar plots representing the frequency of predictive features observed in disseminated (Dis) and non-disseminated (Non-dis) variants. Top: Predictive features with higher frequency in the Dis variants. Bottom: Predictive features with higher frequency in the Non-dis variants. Black dots indicate that a feature carries the corresponding annotation. Feature annotations include: ML feature^a^: Residue labels as defined in ML models. B31seq^b^: Corresponding amino acid residues in the B31 reference sequence. Epitope^c^: Residues predicted to be part of B-cell epitopes by DiscoTope 3.0. Binding^d^ Residues previously reported to be important for binding to host proteins [96]. Surfac^ee^: Residues located at the interface of the plasminogen-OspC-OspC complex, as predicted by AlphaFold3 and ChimeraX. **(C)** AlphaFold-predicted OspC dimer structure. Left: ribbon representation superimposed with the crystal structure (PDB: 1GGQ)[73]. Right: surface views along the 2-fold symmetry axis. **(D)** AlphaFold-predicted structure of the plasminogen-OspC-OspC complex. The five kringle domains of plasminogen are shown in a color gradient (light pink to dark purple). OspC subunits are colored cyan and yellow with ML-predictive residues highlighted in red.

**Fig 5:**
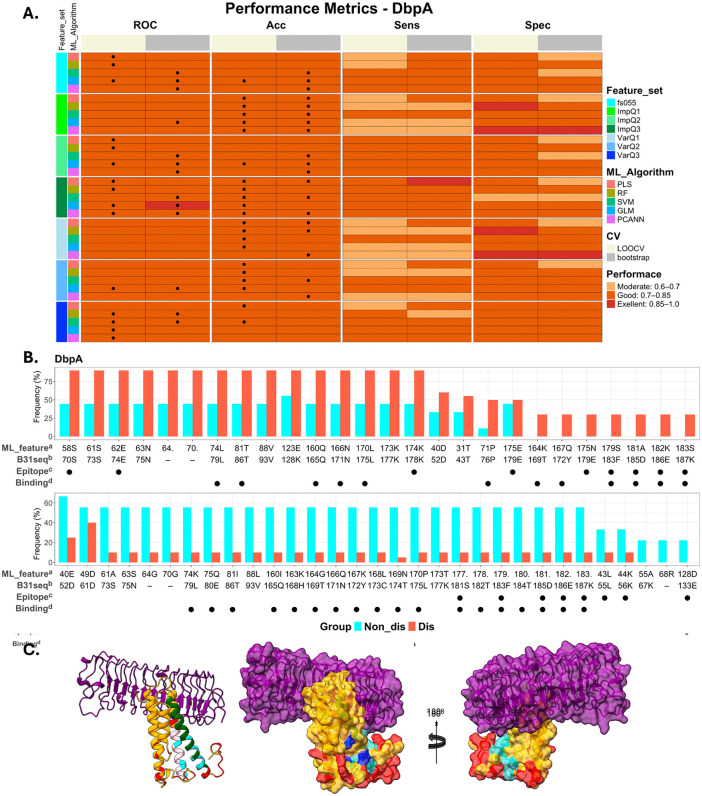
Machine learning model performance and predictive features in DbpA. **(A)** Heatmap displaying average performance values for four key metrics (ROC, Acc, Sens, Spec) evaluated with LOOCV and bootstrap on test datasets. Each cell represents the mean performance value across 100 random train-test splits. Black dots indicate statistically precise estimates (95% CI width < 0.05). Row annotations display the feature sets and ML algorithm as color-coded bar. See figure legend for color code. **(B)** Bar plots representing the frequency of predictive features observed in disseminated (Dis) and non-disseminated (Non-dis) variants. Top: Predictive features with higher frequency in the Dis variants. Bottom: Predictive features with higher frequency in the Non-dis variants. Black dots indicate that a feature carries the corresponding annotation. Feature annotations include: ML feature^a^: Residue labels as defined in ML models. B31seq^b^: Corresponding amino acid residues in the B31 reference sequence. Epitope^c^: Residues predicted to be part of B-cell epitopes by DiscoTope 3.0. Binding^d^ Residues previously reported to be important for binding to host protein [102]. **(C)** AlphaFold-predicted structure of the decorin-DbpA complex. Left: Ribbon representation of the complex. Middle and right: Surface representations from two orientations (rotated 180^o^). DbpA is shown in yellow and decorin in purple. Residues 76–90 and 152–176 of DbpA, known to be important for decorin binding, are highlighted in pink and green, respectively [102]. Red indicates machine learning-identified predictive residues. Cyan marks residues that overlap between ML-predictive sites and known decorin-binding regions. Blue highlights the three conserved Lysines (K82, K163, K170) implicated in glycosaminoglycan binding [100].

**Fig 6:**
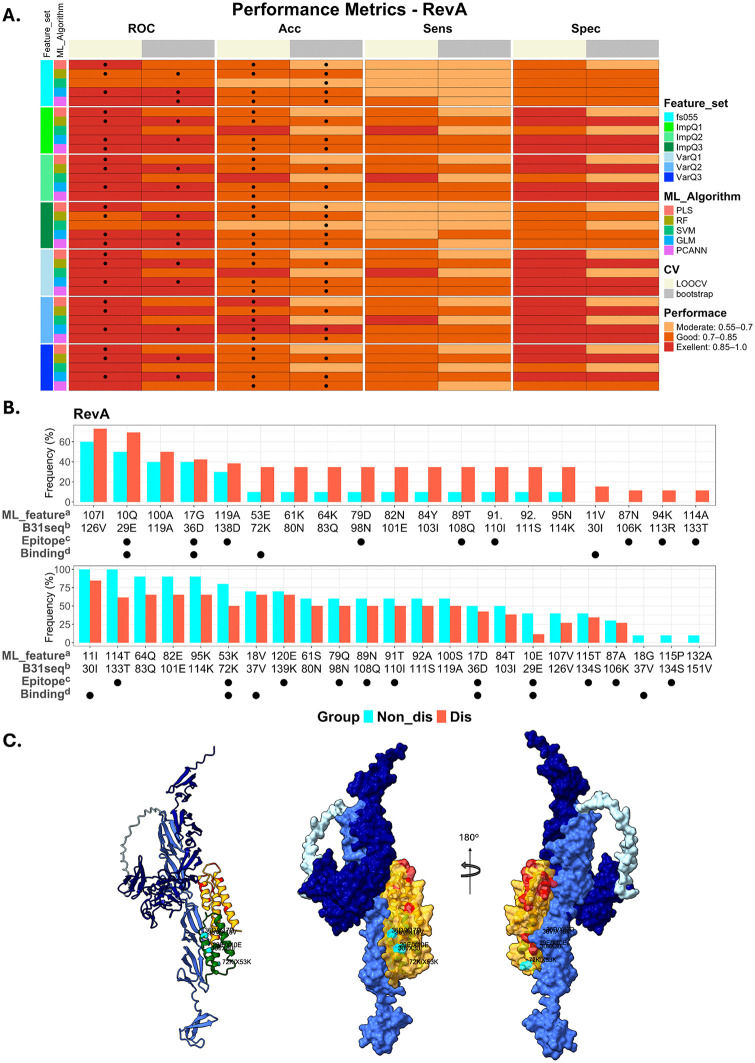
Machine learning model performance and predictive features in DbpA. **(A)** Heatmap displaying average performance values for four key metrics (ROC, Acc, Sens, Spec) evaluated with LOOCV and bootstrap on test datasets. Each cell represents the mean performance value across 100 random train-test splits. Black dots indicate statistically precise estimates (95% CI width < 0.05). Row annotations display the feature sets and ML algorithm as color-coded bar. See figure legend for color code. **(B)** Bar plots representing the frequency of predictive features observed in dissemination (Dis) and non-disseminated (Non-dis) variants. Top: Predictive features with higher frequency in the Dis variants. Bottom: Predictive features with higher frequency in the Non-dis variants. Black dots indicate that a feature carries the corresponding annotation. Feature annotations include: ML feature^a^: Residue labels as defined in ML models; B31seq^b^: Corresponding amino acid residues in the B31 reference sequence; Epitope^c^: Residues predicted to be part of B-cell epitopes by DiscoTope 3.0; Binding^d^: Residues previously reported to be important for binding to host proteins [24]. **(C)** AlphaFold-predicted structure of the fibronectin-RevA complex. Left: Ribbon representation. Middle and right: Surface representations from two orientations (rotated 180^o^). RevA is shown in yellow. Blue and dark blue represent the 30 and 40 kDa regions of fibronectin. Green highlights the N-terminal residues crucial in fibronectin binding [24]. Red indicates machine learning-identified predictive residues. Cyan marks ML-predictive residues within the fibronectin-binding N-terminus.

For each of five proteins (BBK32, DbpA, OspC, P66, and RevA), we developed 70 prediction models by utilizing 2 resampling strategies (LOOCV and Bootstrap) and 5 ML algorithms (GLMnet, PCANN, PLS, RF, and SVM-RBF) to train on 7 different feature subsets derived from one-hot encoded protein sequences (Fig 1). Overall, the ML models built using variant sequences from DbpA, OspC, and RevA demonstrated strong predictive performance across both train and test sets with ROC-AUC values, accuracy, sensitivity, and specificity all exceeding 0.7 (95% CI widths < 0.05) (Figs 4A, 5A, and 6A). BBK32 and P66 models showed weaker performance with ROC-AUC values around 0.6 and exhibited high variability in test sets (S11 Fig). The weaker performance of BBK32 and P66 models compared to DbpA, OspC, and RevA models may reflect several factors: (1) these proteins may have less direct involvement in dissemination mechanisms, (2) their functional variants may be more context-dependent, requiring consideration of additional factors not captured in our current analysis, (3) the highly conserved protein sequence with a concentrated substitution pattern (Table 1) or (4) the sample size may be insufficient to detect subtle but biologically relevant associations for these proteins.

Comprehensive performance metrics, composite score, and rank of all models are provided in S3 Table. Based on the composite scores which equally weighted three key performance metrics (ROC-AUC, sensitivity, and specificity), we identified the optimal model for each protein. PCANN models with ImpQ3 features achieved optimal performance for BBK32 (Bootstrap ROC = 0.613 ± 0.174, LOOCV ROC = 0.630 ± 0.157), DbpA (Bootstrap ROC = 0.842 ± 0.113), and OspC (Bootstrap ROC = 0.934 ± 0.067, LOOCV ROC = 0.908 ± 0.099). For P66 and RevA, different model architectures proved optimal. P66 showed best performance with GLMnet (Bootstrap ROC = 0.682 ± 0.193) and RF (LOOCV ROC = 0.676 ± 0.191) using fs055 features, while RevA achieved highest performance with GLMnet and PLS models using VarQ2 features (Bootstrap ROC = 0.915 ± 0.103, LOOCV ROC = 0.961 ± 0.060). It is important to note that the selection of the best model did not necessarily imply statistically significant superiority over the second-ranked model. S1-S5 Figs illustrated marked heterogenicity in model performance across the seven feature sets and five tested algorithms, with ROC-AUC distributions exhibiting both protein-specific and method-specific patterns. For instance, while no significant differences in ROC-AUC were observed among ML algorithms using feature sets fs055, ImpQ2, ImpQ3, and Var Q3 for OspC, the choice of algorithm had substantially greater impact on model performance with the same types of feature sets for RevA. Notably, complex neural network architectures (PCANN) did not universally outperform linear (GLMnet, PLS) or tree-based (RF) methods. These results underscore the importance of systematic evaluation of multiple algorithms and feature selection strategies, as optimal model-feature combinations are dataset-specific and cannot be predetermined, necessitating comprehensive benchmarking tailored to each classification task.

To assess the impact of different resampling methods on model performance, stability, and generalization, we compared the performance values, coefficients of variation, and train-test differences in LOOCV and Bootstrap models. We showed that LOOCV consistently generated superior performance metrics across all feature sets, algorithms, and proteins, with significantly higher ROC-AUC values, accuracy, and specificity compared to Bootstrap validation (S6 Fig). The LOOCV models demonstrated substantially greater model stability, as evidenced by narrower confidence intervals and reduced variability in performance metrics across the 100 train-test splits (S7 Fig). The train-test performance differences were also significantly smaller in LOOCV models, indicating more consistent generalization and potentially reduced sensitivity to data variation or regularization effects (S8 Fig). These findings demonstrate the importance of resampling strategy selection in small-dataset machine learning applications. LOOCV’s superior performance likely stems from the method’s ability to utilize nearly all available data for training while ensuring each sample is tested exactly once [71]. This is particularly advantageous when working with limited protein variants, where maximizing the use of available data is crucial for robust model development. The Bootstrap method’s poorer performance may be attributed to sampling with replacement, which can lead to some samples being over-represented in training sets while others are excluded, potentially creating artificial patterns or missing important variant information [72]. This sampling bias becomes more pronounced in small datasets where each unique sequence carries significant informational value. The smaller train-test performance differences in LOOCV models indicates reduced overfitting and more realistic performance estimates. We should note that the slight negative gap (approximately -0.05) in Bootstrap models may reflect regularization effects or data variability rather than underfitting.

To assess the agreement between performance estimates derived from LOOCV and Bootstrap methods, we conducted correlation and Bland-Altman analyses across four performance metrics (ROC-AUC, accuracy, sensitivity, and specificity). Correlation analysis revealed excellent concordance for all metrics (ROC-AUC: *r* = 0.932; accuracy: *r* = 0.886; sensitivity: *r* = 0.808; specificity: *r* = 0.903; all *p* < 0.001) (S9 Fig). Complementary Bland-Altman plots confirmed minimal systematic bias between methods. The mean differences between LOOCV and Bootstrap estimates were close to zero for all metrics, and most data points fell within the 95% LOA (S10 Fig)*.* Some protein-specific variations were noted, particularly for RevA, where several measurements showed a positive bias (*i.e.*, LOOCV models exhibited better performance) and more points lay outside the LOA. These outliers suggest that, for RevA, the two resampling methods produced slightly more variable performance estimates compared to other proteins. This likely reflects greater model instability or higher sensitivity of the performance metrics to sample composition for this protein. These findings highlight that the choice of resampling strategy can have a meaningful impact on ML performance estimates.

### 3.3. Model-based important features

We identified the top 20 features in each model and then determined the set of important features or key aa residues for DbpA, OspC, and RevA as described in the Materials and Methods section. 57, 29, and 42 features were selected for DbpA, OspC, and RevA, respectively. Figs 4B, 5B, and 6B show the frequency of the key aa residues in Dis or Non-dis sequences. Several of these residues display distinct prevalence patterns between the two groups, suggesting their role in differentiating dissemination potential. These key aa residues are hereafter referred to as ‘predictive residues’ as they were identified by ML models to predict dissemination potential.

### 3.4. B-cell epitope prediction

To assess the potential immunological relevance of the predictive residues, we predicted B-cell epitopes in the DbpA, OspC, and RevA variants of the B31 strain using AlphaFold3 and DiscoTope 3.0. The DiscoTope scores for individual residues in each protein are listed in S4-S6 Tables. DiscoTope identified 35, 48, and 49 residues within predicted B-cell epitope regions in DbpA, OspC, and RevA, respectively, using a moderate confidence threshold (0.9, recall up to 50%). We found some overlaps between the predicted epitope residues and ML-based predictive residues (Figs 4B, 5B, and 6B).

To evaluate whether the observed overlaps exceeded random expectation, we performed one-sided Fisher’s exact test for each protein. For OspC, the overlap was 11 residues (OR = 3.57, *p* = 0.006), suggesting significant enrichment of dissemination-predictive residues within DiscoTope-predicted epitopes. RevA also exhibited significant enrichment (12 overlapping residues, OR = 2.37, *p* = 0.048). DbpA also had 12 overlapping residues with a potential association (OR = 2.12), although the result was not statistically significant (*p* = 0.058). These results support a biologically meaningful convergence between structural epitope prediction and ML-based residue importance.

### 3.5. Host-pathogen PPI site prediction

DbpA, OspC, and RevA have been well-known to bind to decorin, plasminogen, and fibronectin, respectively. We hypothesized that some of the predictive residues might be located at the interfaces of these PPIs. To explore this, we generated structural models of decorin-DbpA, plasminogen (5-kringle domain)-OspC-OspC, and fibronectin (70 kDa region)-RevA complexes using AlphaFold 3. The predicted Template Modelling (pTM) scores for the decorin-DbpA and plasminogen-OspC-OspC complexes exceeded 0.5, suggesting that the overall folds may be reasonably accurate. However, the pTM of the fibronectin-RevA complex was only 0.3, and the interfacial pTM (ipTM) scores - which assess the relative positioning accuracy between subunits - were low for both decorin-DbpA (0.17) and fibronectin-RevA (0.11) complexes, indicating unreliable interface predictions. The plasminogen-OspC-OspC complex had a moderate ipTM of 0.53, suggesting borderline confidence. Given these limitations, the predicted complex structures were not fully reliable for detailed interface analysis. Nevertheless, mapping the ML-identified predictive residues onto the plasminogen-OspC-OspC model revealed that some may indeed lie within potential interaction sites (Fig 4D). In addition, we generated OspC dimer independently, which showed a high structural similarity to the published crystal structure (PDB: 1GGQ) [73], with an RMSD of 0.257 Å (Fig 4C). The predicted OspC dimer achieved both pTM and ipTM of 0.83. Chimera X identified surface exposed residues based on a solvent-accessible surface area (SASA) threshold of 20 Å^2^. We found that most of the predictive peptides are also surfaced exposed residues, supporting their functional relevance in mediating protein-protein interactions and serving as potential epitopes (Fig 4B-D).

## 4. Discussion

This study represents the first comprehensive ML-based analysis of Bb protein variants and their association with clinical dissemination phenotypes. Through systematic examination of seven known virulence factors across 299 clinical isolates, we identified specific protein variants and aa residues that demonstrate strong predictive capacity for distinguishing between disseminated and localized infections. These findings provide novel insights into the molecular determinants of LD pathogenesis and potential biomarkers for clinical prognosis.

### 4.1. Key findings and their implications

By leveraging WGS data from 299 clinical Bb isolates and employing a combination of correlation analysis and ML models, we demonstrated that variations in several adhesins, particularly DbpA, OspC, and RevA, are significantly associated with the clinical phenotype of dissemination. Our findings underscore the importance of surface-exposed adhesins in the pathogenesis of LD and provide a molecular framework for their continued investigation as potential targets for diagnostics and therapeutics.

Surface-exposed adhesins are integral to Bb’s ability to colonize host tissues, evade immune defenses, and disseminate throughout the host [74,75]. Among the adhesins analyzed, five proteins - BBK32, DbpA, OspC, P66, and RevA - demonstrated significantly strong associations with dissemination phenotypes. The lack of strong association between BB_0406 and OspA in Cramér’s V analysis likely reflects multiple factors beyond biological insignificance. Both proteins are highly conserve (98.4-99.6% identity to the B31 sequence), with <5% of residues substituted, and limited variant diversity reduces the statistical power to detect associations. Additionally, cofounding by unmeasured clinical or demographic factors and potentially indirect or context-dependent roles in dissemination, may further attenuate associations. Importantly, individual variant enrichment analysis revealed that OspA_10 was significantly enriched in localized strains, demonstrating that specific aa substitutions can influence clinical outcomes despite weak overall protein associations.

BBK32 is a fibronectin- and GAG-binding adhesin essential for vascular interactions and joint colonization [25,26,76,77]. RevA, another fibronectin-binding protein with elevated expression during mammalian infection, has been shown to influence dissemination, arthritis severity, and host response [24,78,79]. DbpA, a decorin binding protein that also possesses GAG-binding activity, plays a critical role in tissue colonization and early infection establishment [80–82]. P66, functioning as both an integrin adhesin and porin, has been implicated in endothelial transmigration and tissue invasion [83,84]. OspC is another adhesin crucial for early-stage infection with a dual role in host cell adhesion and evasion of the complement system, exhibiting substantial sequence diversity across Bb strains [85–88]. Certain OspC types are preferentially associated with invasive phenotypes [10,89–91]. Comparative analysis of BBK32 orthologues from different LB species reveals that both *B. garinii* and *B. afzelii* variants bind C1 and C1r complement components with high affinity similar to Bb BBK32 though only the *B. afzelii* BBK32 orthologue (BGD19) demonstrates potent inhibition of the classical complement pathway like Bb BBK32 [92]. Similarly, DbpA variants across different LB species have been shown to affect spirochetes binding to decorin, dermatan sulfate, and mammalian cells, as well as influence tissue tropism in mice [93,94]. Our study demonstrated that within Bb, there are certain variants of these proteins exclusively associated with either localized or disseminated infections in humans. While most variants with exclusive outcomes were represented by a very small sample size and may reflect sampling limitations, they may still represent authentic biological associations reflecting the diversity of Bb isolates sampled over the 30-year collection period. In overall, the results further support potential roles of these proteins in tissue invasion and provide essential reference points for studying interspecies variations in pathogenesis mechanisms and clinical outcomes.

We successfully built robust ML models capable of distinguishing protein sequences of DbpA, OspC, and RevA that are potentially associated with dissemination, with all performance metrics exceeding 0.7. This represents a novel computational approach to predicting Bb virulence potential based solely on protein sequence data. The feature importance analysis revealed 57, 29, and 42 key predictive residues for DbpA, OspC, and RevA, respectively. These residues are considered strongly associated with differential dissemination capacity, though their causal roles in determining virulence potential require further validation. Our findings provide a molecular-level hypothesis for future investigation of Bb pathogenesis mechanisms.

### 4.2. Biological relevance of predictive peptides

The convergence between our ML-identified predictive residues and computationally predicted B-cell epitopes provides compelling evidence for the biological relevance of our findings. The significant enrichment of predictive residues within predicted epitope regions for OspC (OR = 3.57, *p* = 0.006) and RevA (OR = 2.37, *p* = 0.048) suggests that immune recognition of these specific residues may play a crucial role in determining disease progression. Variants with epitope residues that are less immunogenic or exhibit altered antigenicity may have enhanced capacity to disseminate by avoiding rapid immune clearance.

These results are particularly noteworthy when compared to previously characterized epitopes in these proteins. The Immune Epitope Database (IEDB) (www.iedb.org) is a comprehensive resource that catalogs experimentally validated immune epitopes from infectious, allergic, and autoimmune disease and transplant rejection studies in humans, non-human primates, and other animal species [95]. We confirmed that many of the ML-identified predictive residues and DiscoTope-predicted epitopes in OspC and DbpA lie within the linear B-cell epitopes extracted from IEDB (S12 Fig). There was no available IEDB data for RevA. A previous study reported that the human B-cell epitope regions on B31 OspC (type A), including residues _B31_71-86, _B31_104-118, _B31_156-171, and _B31_184-190, are within the binding sites of OspC and mouse B5 antibody [96]. B5 is the only mouse OspC-specific monoclonal IgG2a antibody (MAb) that has been demonstrated to protect mice against Bb challenge via either needle injection or tick infection [97–99]. Interestingly, some of the OspC predictive residues within the regions _B31_71-86 and _B31_104-118 were observed at least two-fold more frequently in Non-dis variants: X61Q(_B31_79K), X67N(_B31_85N), X88A(_B31_106T), and X95N(_B31_113D) (Fig 4B). Conversely, other predictive residues within these regions were observed at least two-fold more frequently in Dis variants: X56.(_B31_74A), X65N(_B31_83Q), X99K(_B31_116K), and X100N(_B31_117N) (Fig 4B). Collectively, these findings underscore the potential role of immune-targeted residues in influencing pathogen dissemination, reinforcing the relevance of our predictive approach to identifying biologically meaningful targets.

We hypothesized that predictive residues play roles in host-pathogen interactions. Unfortunately, all the host-pathogen protein complex structures predicted by AlphaFold 3 had very low ipTM values, which implied failed prediction in the relative orientation of interacting partners. Although this limits the identification of specific interface residues for each complex, we observed some of the OspC predictive residues located within potential interaction sites of plasminogen-OspC-OspC (Fig 4D). In addition, although the dimer interface of OspC buried approximately 22% of the accessible surface area of each unit [73], most of the OspC predictive residues were located outside this buried region and overlap with Chimera X predicted surface-exposed residues (Fig 4C). This result indicates their potential availability for interactions with host proteins and/or recognition by the immune system, suggesting a potential role in disease dissemination.

For DbpA, several residues including Lysines _B31_82, _B31_163, and _B31_170, as well as the C-terminal tail have been characterized to be important for GAG binding [100,101]. The three Lysines are conserved in all DbpA variants in our study. The peptide sequence _B31_76-90 was shown to be crucial for decorin-binding while peptide sequence _B31_152-176 retained ligand binding activity by maintaining the appropriate decorin-binding conformation [102]. While these experimentally validated peptide regions did not overlap with the binding site in our predicted decorin-DbpA structure - likely due to the small ipTM value - several of our ML-based predictive residues are located within these regions and the C-terminal tail (Fig 5B and C). A similar pattern was observed with RevA, where the first 60 aa residues on the N-terminus, previously shown to be essential for fibronectin binding [24], did not overlap with the binding site on the predicted fibronectin-RevA structure (Fig 6B and C). Nevertheless, our ML models identified several N-terminal residues as important for distinguishing potentially disseminated variants. Collectively, these results support the biological relevance of our predictive residues and their potential roles in mediating host-pathogen interactions crucial for disease dissemination.

### 4.3. Study limitation and future directions

While our computational approach offers valuable insights, several limitations must be acknowledged. First, the relatively small dataset size and class imbalance required the use of SMOTE oversampling, which may introduce artificial patterns in the data. Second, we simplified the classification of variants into just two classes based on any present/absence of the variants in disseminated isolates. This binary classification approach may oversimplify dissemination as a discrete trait when it likely exists on a continuous spectrum, and sequences labeled as “Non-dis” may reflect incomplete clinical sampling rather than true lack of dissemination capacity.

Third, although our ensemble ML framework with nested cross-validation approach achieved robust predictive performance, limitations remain in both evaluation and interpretability. several of the implemented models offer limited direct interpretability. While our repeated random splitting offers stable performance estimates and likely captures diverse partitions, explicit sequence similarity-based splitting would more rigorously validate transferability. Additionally, while GLMnet enables sparse feature selection, the other methods (PLS, RF, SVM-RBF, and PCANN) limit direct interpretability of individual residue variation contribution and their interactions. Future studies could employ similarity-aware data partitioning and explore interpretable approaches such as a sparse logistic regression with hierarchical interaction methods (e.g. hierNet) to strengthen both evaluation rigor and biological interpretation.

Fourth, while our structural predictions provide valuable insights, the low ipTM scores for the protein complexes limit the reliability of interface analyses. Future studies incorporating experimental validation of predicted interaction sites through mutagenesis binding assays and infection models will be necessary to confirm the functional impacts of identified residues.

Fifth, our analysis focused on individual aa variations within a single protein from a limited set of known virulence factors, potentially overlooking epistatic interactions within proteins, synergistic effects across multiple proteins, and important determinants elsewhere in the Bb genome. Future studies should investigate combinatorial variations both within and across proteins, assess co-occurrence patterns among multiple proteins in disseminated strains, and expand toward broader genome-wide approaches to more comprehensively define determinants of dissemination capacity. In addition, because the study relied on previously published data with heterogeneous clinical metadata, collected over a 30-year period, we did not explicitly control for potential confounders such as bacterial population structure, geographic sampling biases, patient demographics, or evolving diagnostic criteria, all of which may influence observed associations.

## 5. Conclusions

This study demonstrates the power of ML approaches in identifying biologically relevant patterns in pathogen genomic data. The strong predictive performance of DbpA, OspC, and RevA variant-based models, combined with the immunological relevance of predictive residues, provides a foundation for both mechanistic understanding and clinical application. The methodological framework developed here may also be applicable to other bacterial pathogens where strain-level variation influences disease outcomes.

## Supporting information

S1 Fig AUC-ROC comparison of machine learning algorithms for BBK32 models. Statistical significance of pairwise t-test comparisons between algorithms is indicated above each panel. ****p < 0.0001, ***p < 0.001, **p < 0.01, *p < 0.05.
(TIF)

S2 Fig AUC-ROC comparison of machine learning algorithms for DbpA models. Statistical significance of pairwise comparisons between algorithms is indicated above each panel. ****p < 0.0001, ***p < 0.001, **p < 0.01, *p < 0.05.
(TIF)

S3 Fig AUC-ROC comparison of machine learning algorithms for OspC models. Statistical significance of pairwise t-test comparisons between algorithms is indicated above each panel. ****p < 0.0001, ***p < 0.001, **p < 0.01, *p < 0.05.
(TIF)

S4 Fig AUC-ROC comparison of machine learning algorithms for P66 models. Statistical significance of t-test pairwise comparisons between algorithms is indicated above each panel. ****p < 0.0001, ***p < 0.001, **p < 0.01, *p < 0.05.
(TIF)

S5 Fig AUC-ROC comparison of machine learning algorithms for RevA models. Statistical significance of pairwise t-test comparisons between algorithms is indicated above each panel. ****p < 0.0001, ***p < 0.001, **p < 0.01, *p < 0.05.
(TIF)

S6 Fig Performance comparison between LOOCV and bootstrap models across all proteins and feature sets. Statistical significance of pairwise t-test comparisons between algorithms is indicated above each panel. ***p < 0.001, **p < 0.01.
(TIF)

S7 Fig Model stability comparison between LOOCV and bootstrap models across all proteins and feature sets. Statistical significance of pairwise t-test comparisons between algorithms is indicated above each panel. ***p < 0.001, *p < 0.05.
(TIF)

S8 Fig Generalization gap plots of LOOCV and bootstrap models across all protein and feature sets. Statistical significance of pairwise t-test comparisons between algorithms is indicated above each panel. ***p < 0.001.
(TIF)

S9 Fig Correlation between LOOCV and bootstrap models across performance metrics. Each point represents a gene-specific model, color-coded by gene (BBK32: orange, DbpA: blue, OspC: red, P66: gray, RevA: green). The solid black line represents the regression line, with Pearson correlation coefficients (r) and p-values displayed.
(TIF)

S10 Fig Bland-Altman plots assessing agreements between LOOCV and bootstrap resampling methods. Each point represents a gene-specific model evaluation, color-coded by gene (BBK32: orange, DbpA: blue, OspC: red, P66: gray, RevA: green). The solid blue line indicates mean bias (zero line represents perfect agreement), and dashed red lines represent 95% limits of agreement (±1.96 SD).
(TIF)

S11 Fig Machine learning model performance and predictive features in (A) BBK32 and (B) P66. Heatmap displaying average performance values for four key metrics (ROC, Acc, Sens, Spec) evaluated with LOOCV and bootstrap on test datasets. Each cell represents the mean performance value across 100 random train-test splits. Black dots indicate statistically precise estimates (95% CI width < 0.05). Row annotations display the feature sets and ML algorithm as color coded bar. See figure legend for color code.
(TIF)

S12 Fig Positive immune assay counts mapped to DbpA and OspC sequence. Data collected from IEDB ImmunoBrowser. Red line indicates the number of positive immune assays mapped to each amino acid position along the protein sequences. Green triangles represent DiscoTope-predicted discontinuous B-cell epitopes, and purple circles indicate machine learning-predicted residues.
(TIF)

S1 Table List of protein variants. (XLSX)

S2 Table Numbers of features in input dataset for each model. (XLSX)

S3 Table Model performace in train and test sets (Means ± SD (95%CI), best models are highlighted). (XLSX)

S4 Table DiscoTope results of dbpA. (XLSX)

S5 Table DiscoTope results of OspC. (XLSX)

S6 Table DiscoTope results of RevA. (XLSX)
