# Supplementary material for: Machine learning-driven identification of virulence determinants in Borrelia burgdorferi associated with human dissemination
Source: PLoS Comput Biol. 2026 Jun 17;22(6):e1014407. doi: 10.1371/journal.pcbi.1014407 (PMC13293517; doi:10.1371/journal.pcbi.1014407)
Supplement: Attachment — Submitted filename: Review PCOMPBIOL-D-25-02087.pdf [file pcbi.1014407.s019.pdf]

Review PCOMPBIOL-D-25-02087

Machine learning-driven identification of virulence determinants in *Borrelia burgdorferi* associated with human dissemination

Summary:

The authors performed a detailed analysis of protein sequences from infectious isolates of *B. burgdorferi.* Using publicly available whole-genome sequencing data, they focused on seven surface lipoproteins implicated in the pathogenesis of lyme disease caused by *B. burgdorferi,* and trained machine learning models able to classify protein sequences associated with disseminated infections.

Overall, the manuscript is very well-structured, detailed, and transparent about potential limitations. Nonetheless, there are some areas where its strength could be improved.

Major comments:

The main figures in the manuscript could deliver information in a better way, some of which is currently relegated to the Supporting Information. Specifically:

Figure 1 could be problematic to extract information from. The predominant message of this figure seems to be the percentage of dissemination-associated isolates per sequence for each protein. However, the individual sequences are difficult to distinguish, and this distinction does not seem to be relevant in the text. Therefore, why not reduce the information to one plot per protein (e.g., pie chart or heatmap) and combine with the more informative Figure 2? Alternatively, if the goal is to highlight the sequences exclusively associated with dissemination, they could be plotted separately, and the other sequences represented showing that most were observed in both disseminated and non-disseminated infection strains.

Similarly, Figure 3 contains details which may be unnecessary for the reader. The main conclusion here is the combined predictive performance metrics of ML models per protein. This could be simplified or broken down into subplots relevant to either the feature set, the ML algorithm, or the performance metric. Currently, the figure makes it difficult to distinguish the individual ML model performance without considerable effort. Additionally, the intensity scale should be labeled.

Information from Figure 4 is very granular. The sequences could be sorted by the '+'-values in each category and color-coded, to make it easier for the reader to interpret. The y-axis should also be labeled.

Figures S11-S13 provide very useful visualization for the entire subchapter 3.5 (lines 364-386 of the Results section) and demonstrate actual biological significance of ML modeling to residues predictive in protein-protein interactions. I believe they should be moved to the main text, as they constitute major conclusions inferred from the analysis.

Minor comments:

p.4 line 61 "*Several studies have also reported the binding of surface-exposed lipoproteins to the host extracellular matrix (ECM) such as fibronectin (24–26), glycosaminoglycans (GAGs) (27–29), and plasminogen (30–33).*"
This sentence could be restructured. At first glance, it seems to mean fibronectin, GAGs, and plasminogen are the lipoproteins, and not parts of the ECM.

p.5 line 82 *The surface-exposed proteins examined (BBK32, DbpA, OspA, OspC, P66, and RevA) were selected based on their established roles in host cell adhesion, immune evasion, and tissue invasion processes critical to bacterial dissemination.*
Is there a reason why BB0406 is not mentioned here? As far as I understand, it is an outer membrane protein in Bb paralogous to BB0405 and considered less important than it for infectivity (doi: 10.1128/IAI.00803-16) but this is not discussed in the introduction.

p.12 line 239 "(…), we identified between 4 and 36 unique variants per protein."
What do the authors mean by protein "variants"? This word is often seen in the context of protein isoforms but here it seems meaning unique amino acid sequences with respect to isolate. If so, it could be useful to state this explicitly, especially since the word "variants" is then used extensively.

p. 18 line 369, Abbreviation "TM" is never explained

Other points:

There are several small grammatical errors and typos throughout the manuscript which do not change the general meaning but should be addressed before publication.

p.3 line 48 "…technical challenges for sequencing, assembly, and analysis the LB genome,
ultimately limiting…"
missing "of"

p.6 line 100 "Sequences were aligned and removed signal sequences prior to feature calculations."
signal sequences "were" removed

p. 10 line 188 "Generalization gap, defined as the difference between train and test performance (Bias
= Train – Test), quantified overfitting (positive values) or underfitting (negative values) with comparison conducted across all stratification levels."
missing "was"

p.13 line 259 “*BB_0406 and OspA did not exhibit strong associations with clinical phenotypes, which potentially due to their low variant diversity.*”
missing “is/was” or remove “which”

p. 17 line 344 “There were 57, 29, and 42 features were selected for DbpA, OspC, and RevA, respectively.”
Unnecessary “there were”

p. 18 line 371 “*suggesting that the overall folds may reasonably accurate”*
missing “be”
