# Supplementary material for: Machine learning-driven identification of virulence determinants in Borrelia burgdorferi associated with human dissemination
Source: PLoS Comput Biol. 2026 Jun 17;22(6):e1014407. doi: 10.1371/journal.pcbi.1014407 (PMC13293517; doi:10.1371/journal.pcbi.1014407)
Supplement: Attachment — Submitted filename: PCOMPBIOL-D-25-02087_Reviewer comments.pdf [file pcbi.1014407.s020.pdf]

PCOMPBIOL-D-25-02087

Reviewer comments

This manuscript presents a comprehensive machine learning (ML) framework to identify amino acid (AA) residues within well-characterized *Borrelia burgdorferi* virulence factors that predict severe human dissemination outcomes using genomic data from 299 clinical isolates. The study extends beyond the ML approach by integrating B-cell epitope prediction and host-pathogen protein-protein interaction analyses to assess the biological relevance of the identified residues. The work addresses an important public health problem, uses appropriate methodology, and provides potentially impactful results. However, several aspects require clarification and revision to improve rigor, interpretation, and presentation.

Please consider the following remarks.

1. Several terms are used ambiguously, which may cause confusion. Consistent and precise terminology should be applied for scientific accuracy. Terms “protein variants” (e.g., Line 17) or “protein sequences” (e.g., Line 122/433/Fig1 caption) or “predictive peptides” are potentially misleading, as the analyses involve translated AA sequences derived from virulence factor genes identified from WGS data rather than experimentally validated protein variants (e.g. putative proteins encoded by their respective genes or translated AA sequences). Additionally, the term “sequence” without specifying gene or AA sequences (e.g., Line 65/87/97), and phrases like “predicted predictive and epitope residues” may also be confusing.

2. Line 22-26: Please report the number of residues identified by ML for each protein (e.g., 57 for DbpA) and specify their overlap with residues predicted by B-cell epitope analysis.

3. Since the primary aim of this study is to present a computational framework, the manuscript should briefly discuss prior approaches (e.g., traditional GWAS or statistic methods) and their limitations, to provide context for the advantages of an ML-based approach.

4. The manuscript lacks sufficient details about the 299 clinical isolates (e.g., regions/countries, sampling period), making it difficult to interpret the representativeness and distribution. Please provide a brief description of the dataset, including the number of “Dis” *vs.* “Non-dis” cases, in the Methods (and abstract, if possible), even if this is a secondary dataset.

5. Line 122-123: Please provide the lengths of each gene (bp) and putative protein (AA) and indicate the remaining AAs after signal sequence removal. Also justify the rationale for removing signal sequence. If the gap character (Line 126) represents indels, please state this explicitly to avoid ambiguity and improve reproducibility.

6. Please list all software and tools used (e.g., Cramér’s V, sequence alignment/editing, pairwise distance calculations) and specify the parameters for DiscoTope analysis, even if default settings were applied, to ensure reproducibility.

7. Line 259-261/406-408: Please clarify how Cramér’s V analysis handles sparse contingency tables. The weaker associations of BB_0406 and OspA may reflect factors beyond low variant diversity, such as confounding clinical/demographic variables or a less direct role in

dissemination. Discussing these possibilities would provide a more nuanced interpretation. Even if variants (as collective AA compositions) show weak associations, specific AA substitutions could still correlate with clinical outcomes.

8. Line 100-102: It is unclear what "distance" represents. Please report pairwise AA percent identity ranges for each putative protein relative to B31, as well as among all unique variants, to illustrate sequence diversity (e.g., percent identity with conserved counts). This would clarify whether observed variant counts result from multiple substitutions at a few positions or fewer substitutions spread across many sites (e.g., BB_0406).

9. Line 242-251/425-429: Sample size should be considered when interpreting associations for variants with exclusive outcomes. With only a few cases, the observed exclusivity could occur by chance, reflecting under- or non-representative sampling and potentially introducing bias. Reporting the number of cases for these variants would provide important context and improve the interpretation.

10. Line 458-459/Fig 4: It is unclear whether the AA positions reported in the cited study (e.g., 71-86, 104-118,) directly correspond to the ML-predicted residues in this study, as alignment differences and indels can shift numbering. For example, if residues 71-86 in the cited study were based on B31, the ML-predicted residue X61K/Q may fall within that region. Using AA positions aligned to a well-established reference sequence (e.g., B31) would enable accurate comparisons across studies and improve the interpretability of residue-level analyses (e.g., X61K associated with "Dis" *vs.* X61Q with "Non-dis").

11. The study relies on secondary data, which is a potential limitation. Are there other risk factors that could act as confounders, such as population structure of *B. burgdorferi* isolates or differences in patient demographics? If so, please discuss these possibilities and, if feasible, describe how such confounding factors were assessed or controlled for in the analyses.

Minor Comments:

1. Consistent abbreviation should be maintained throughout the manuscript (e.g., "Bb" for *B. burgdorferi vs. B. burgdorferi sensu stricto*, although both refer to the same species). Also, Lyme borreliosis (LB) is the disease and should not be used as a collective term for the *Borrelia* complex (e.g., Line 34/41).

2. Line 103-106: Consider providing examples of sequences identifiers (e.g., BB_0406_1 or A-1) and listing all OspC types included in the study for clarity.

3. Figures require formatting improvements for clarity and consistency. Specifically:
    a. Ensure all axes are clearly labeled (Fig1/2/4),
    b. Place shared legends for multi-panel figures in a clear, consistent location (e.g., below the panels in Fig1)
    c. Consider providing categorical legends for heatmaps to clarify color coding (Fig3).
    d. In Fig4, the x-axis label "binding" is used inconsistently across different protein panels; please clarify and standardize the labeling.

4. A few references are missing (Line 35/365-366), including for NCBI BioProject PRJNA923804, the B31 reference strain, and the OspC crystal structure (PDB: 1GGQ).

5. Please revise minor typographical or formatting errors in Line 59/60/217/226/475/486/Fig2 caption (e.g., surface exposed, Borrelia protein variants, surface-exposed residue). BB_0406 is also missing in Line 81-82.
