# Supplementary material for: Machine learning-driven identification of virulence determinants in Borrelia burgdorferi associated with human dissemination
Source: PLoS Comput Biol. 2026 Jun 17;22(6):e1014407. doi: 10.1371/journal.pcbi.1014407 (PMC13293517; doi:10.1371/journal.pcbi.1014407)
Supplement: Attachment — Submitted filename: Reviewer 1 comments.pdf [file pcbi.1014407.s021.pdf]

Overall comments:
In this manuscript, Nguyen & Brissette aim to find molecular features that are determinants of disseminating infections in Lyme disease. They approach this task by training various ML models to classify protein variants as coming from at least one strain associated with a disseminating infection. They show that the variants highlighted by these ML models are associated with host dynamics. This is an important task and I commend the authors for tackling this challenge.

However I do have some concerns:

1. In the Methods section (lines 108-109) the authors describe that protein variants are assigned to the the Disseminating (Dis) or Non-Disseminating (Non-Dis) class if a variant is found in *any* disseminating strain. Does this mean that if all strains but one are non-disseminating, the proteins variants are still classified as disseminating? If so, this would lead to an artificial overrepresentation of the Dis class and lead to erroneous associations, as the distribution of the classes no longer reflects the actual distribution. It's important for the authors to discuss and justify this decision and show that it does not lead to erroneous associations.

2. Given that the Dis class is more common than the Non-Dis, conventional machine learning practice would follow a strategy where the Non-Dis class is encoded as the positive class. At the moment, it is unclear in the manuscript whether this is what the authors did, and if so, how were variants determined to be determinants of the Dis class, and not of the Non-Dis class.

3. The ML methods chosen by the authors are known for their good out-of-box performance, but few of them are easily interpretable. Why did the authors not explore the use of (sparse) logistic regression and evaluate the contribution of individual variants by looking at model coefficients. One could even evaluate a method such as hierNet (https://cran.r-project.org/web/packages/hierNet/hierNet.pdf) to measure the contribution of the combination of different variants. It is unclear which GLM the authors used in this task.

4. During cross validation and bootstrapping it seems like the training and testing sets are constructed somewhat randomly. In principle, there should also be a validation set where model hyperparameter configurations can be selected and finally compared in the test set. Furthermore, a more realistic evaluation of the models would split the protein sequences into training, testing and validation based on protein sequence similarity. In other words, proteins in training should be distinct from those in testing and validation. This would confirm the generalisability of the proposed variants to discriminate between Dis and Non-Dis.

5. I am still unclear about the strength of the association between the proposed variants and the Dis infections. In Figure 4 it seems like many of the proposed variants (particularly for OspC and RevA) are actually more prevalent in the non-Dis strains (it is unclear what the X-axis is indicating), even when the variant is found in the B-cell epitope region. This is somewhat less convincing than what is stated by the authors: „Our findings reveal that specific aa residues within these surface exposed proteins serve as critical determinants of dissemination capacity" (lines

433-445). None of the variants found is *only* found in the Dis class, indeed some are even more prevalent in Non-Dis strains.

6. At the moment, the authors explore how variants in single proteins are associated with Dis capacity. The authors could explore the effect of combined variants within the same protein sequence, as well as the co-presence of different variants in the surface proteins in the same strain. Indeed, it might be interesting to know in how many of the Dis strains the proposed protein variants co-exist.

Furthermore the following clarifications:

1. How many of the collection of 299 strains were initially associated with being Dis or Non-Dis?
2. How many unique sequences were found for each protein variant? How many variants of each protein were determined to be Dis, or non-Dis? Does this deviate substantially from the strains class distribution?
3. More details should be given for the selected ML methods. Why were they selected and how were they implemented?
4. More details should be given for the Variable Importance scores. How were these calculated exactly? Is each feature evaluated for its ability to discriminate between classes?
5. To evaluate the tradeoff between Specificity and Sensitivity the authors might be interested in exploring the Precision-Recall curve AUC, which is perhaps more informative in class imbalance regimes.
6. It is unclear to me how residues are removed during pre-processing (lines 128-130). Does this mean that if the same AA is found in the same position across all sequences, then this position is not considered?

Comments on code:
The provided code seems complete and is well documented. However I was unable to run the individual scripts due to various errors. For example:
When I run OHE_code.R it seems 0 sequences are processed correctly
When I run prepare Create_Input_data.R it seems like the filterVarImp function is not defined.

My sessionInfo:

R version 4.5.1 (2025-06-13)
Platform: x86_64-apple-darwin20
Running under: macOS Tahoe 26.1

Matrix products: default
BLAS:
/System/Library/Frameworks/Accelerate.framework/Versions/A/Frameworks/vecLib.framework/Versions/A/libBLAS.dylib
LAPACK:
/Library/Frameworks/R.framework/Versions/4.5-x86_64/Resources/lib/libRlapack.dylib; LAPACK version 3.12.1

attached base packages:

```
[1] stats4    stats     graphics  grDevices utils     datasets  methods   base

other attached packages:
 [1] readxl_1.4.5          Biostrings_2.76.0     GenomeInfoDb_1.44.0 XVector_0.48.0
 [5] IRanges_2.42.0        S4Vectors_0.46.0      BiocGenerics_0.54.0 generics_0.1.4
 [9] Peptides_2.4.6        caret_7.0-1           lattice_0.22-7      ggplot2_3.5.2

loaded via a namespace (and not attached):
 [1] gtable_0.3.6            recipes_1.3.1           vctrs_0.6.5
tools_4.5.1
 [5] parallel_4.5.1          tibble_3.3.0            pkgconfig_2.0.3
ModelMetrics_1.2.2.2
 [9] Matrix_1.7-3            data.table_1.17.6       RColorBrewer_1.1-3
lifecycle_1.0.4
[13] GenomeInfoDbData_1.2.14 compiler_4.5.1          farver_2.1.2
stringr_1.5.1
[17] codetools_0.2-20        class_7.3-23            prodlim_2025.04.28
crayon_1.5.3
[21] pillar_1.11.0           MASS_7.3-65             gower_1.0.2
iterators_1.0.14
[25] rpart_4.1.24            foreach_1.5.2           nlme_3.1-168
parallelly_1.45.0
[29] lava_1.8.1              tidyselect_1.2.1        digest_0.6.37
stringi_1.8.7
[33] future_1.58.0           dplyr_1.1.4             reshape2_1.4.4
purrr_1.0.4
[37] listenv_0.9.1           splines_4.5.1           grid_4.5.1              cli_3.6.5
[41] magrittr_2.0.3          survival_3.8-3          future.apply_1.20.0
withr_3.0.2
[45] scales_1.4.0            UCSC.utils_1.4.0        lubridate_1.9.4
timechange_0.3.0
[49] httr_1.4.7              globals_0.18.0          nnet_7.3-20
timeDate_4041.110
[53] cellranger_1.1.0        hardhat_1.4.1           rlang_1.1.6
Rcpp_1.1.0
[57] glue_1.8.0              pROC_1.19.0.1           ipred_0.9-15
rstudioapi_0.17.1
[61] jsonlite_2.0.0          R6_2.6.1                plyr_1.8.9
```
