# Supplementary material for: Machine learning-driven identification of virulence determinants in Borrelia burgdorferi associated with human dissemination
Source: PLoS Comput Biol. 2026 Jun 17;22(6):e1014407. doi: 10.1371/journal.pcbi.1014407 (PMC13293517; doi:10.1371/journal.pcbi.1014407)
Supplement: Attachment — Submitted filename: Response to reviewers.docx [file pcbi.1014407.s023.docx]

We appreciate the reviewer's careful evaluation and valuable suggestions for improving our manuscript. This document provides point-by-point responses to all reviewer comments. Original reviewer comments appear in black text, followed by our responses in red text. Line numbers refer to the revised manuscript.

**# Reviewer 1 comments**

Overall comments:

In this manuscript, Nguyen & Brissette aim to find molecular features that are determinants of disseminating infections in Lyme disease. They approach this task by training various ML models to classify protein variants as coming from at least one strain associated with a disseminating infection. They show that the variants highlighted by these ML models are associated with host dynamics. This is an important task and I commend the authors for tackling this challenge.

However I do have some concerns:

1. In the Methods section (lines 108-109) the authors describe that protein variants are assigned to the the Disseminating (Dis) or Non-Disseminating (Non-Dis) class if a variant is found in *any* disseminating strain. Does this mean that if all strains but one are non-disseminating, the proteins variants are still classified as disseminating? If so, this would lead to an artificial overrepresentation of the Dis class and lead to erroneous associations, as the distribution of the classes no longer reflects the actual distribution. It’s important for the authors to discuss and justify this decision and show that it does not lead to erroneous associations

It is correct that we assigned protein variants to the Dis class even if all but one strain were non-disseminating. We agree that this permissive classification strategy would lead to overrepresentation of the Dis class. This trade-off was made to ensure that all potentially dissemination-relevant variants are captured and to support our analytical goal of identifying candidate amino acid residues that may promote or inhibit dissemination. We also recognize the limitations of our data, including simplified binary classification and small or uneven sample sizes for some variants, as noted in the Discussion. Despite these limitations, the selected important features (i.e. predictive residues) from our ML models may have biological relevance, as demonstrated as possible B-cell epitopes and host-protein binding sites.

On the other hand, the Cramer’s V analysis was performed at the isolate level using contingency tables where each cell represents the actual counts of isolates carrying a specific variant with a specific clinical outcome. This analysis reflects the overall associations between proteins and dissemination, independent of the variant-level Dis or Non-dis assignment. We have also added the standardized residuals analysis to identify which specific variants drive the association. The analysis reveals which variants are significantly enriched in Disseminated and Localized phenotypes (Table 2).

2. Given that the Dis class is more common than the Non-Dis, conventional machine learning practice would follow a strategy where the Non-Dis class is encoded as the positive class. At the moment, it is unclear in the manuscript whether this is what the authors did, and if so, how were variants determined to be determinants of the Dis class, and not of the Non-Dis class.

In our ML models, we correctly labeled the Dis as positive and Non-dis as negative class. Thus, the sensitivity and specificity score correctly reflects the true positive rate for Dis class and true negative rate for Non-dis class. We have specified this in the revised manuscript (lines 251, 252).

We identified the important features based on variable importance (VIP) scores, which were computed using the varImp() function from the caret R package. All the five algorithms were applied for a binary classification problem, and varImp() returned a single overall score per feature reflecting the overall contribution of each feature to discriminating between Dis and Non-dis class rather than separate positive-class-specific values. For GLMnet, VIP was derived from the absolute values of the coefficients at the optimal regularization parameter. For PLS, VIP was based on weighted sums of absolute regression coefficients across latent components, where weights are proportional to the reduction sums of squares. For Random Forest (RF), VIP is computed from the mean decrease in Gini impurity across all trees in the ensemble. For SVM (radial kernel) and Principal Component Analysis Neuron Network (PCANN), VIP is estimated using model-agnostic sensitivity measures, providing overall importance per feature. We have added this details in the revised manuscript (lines 282-291).

3. The ML methods chosen by the authors are known for their good out-of-box performance, but few of them are easily interpretable. Why did the authors not explore the use of (sparse) logistic regression and evaluate the contribution of individual variants by looking at model coefficients. One could even evaluate a method such as hierNet (https://cran.r-project.org/web/packages/hierNet/hierNet.pdf) to measure the contribution of the combination of different variants. It is unclear which GLM the authors used in this task.

We thank the reviewer for this insightful critique on interpretability. We acknowledge that while the selected algorithms offer strong predictive performance, they are limited in directly interpreting individual and interactive effects. For GLM, we used elastic net regularization (GLMnet), which balances L1 (Lasso) and L2 (Ridge) penalties through a tuned α parameter. This hybrid approach enables feature selection by driving some coefficients to zero (as in sparse logistic regression), improving interpretability and reducing overfitting. Concurrently, it stabilizes coefficient estimates through Ridge shrinkage, offering flexibility and robustness in high-dimensional data. We also employed PLS, RF, SVM, and PCANN, which leverage complementary strengths in capturing linear, non-linear, and complex patterns, enabling robust important feature discovery across different learning paradigms. We agree that hierarchical interaction methods such as hierNet could further elucidate interactions among variants and have noted this as a promising direction for future work (lines 732-734).

4. During cross validation and bootstrapping it seems like the training and testing sets are constructed somewhat randomly. In principle, there should also be a validation set where model hyperparameter configurations can be selected and finally compared in the test set. Furthermore, a more realistic evaluation of the models would split the protein sequences into training, testing and validation based on protein sequence similarity. In other words, proteins in training should be distinct from those in testing and validation. This would confirm the generalisability of the proposed variants to discriminate between Dis and Non-Dis.

We appreciate the reviewer’s clarification regarding the three-way data split (training, validation, and test). Our nested cross-validation strategy was designed to implement this structure while maximizing the use of the available data. Within each training fold (75% or data), we created explicit validation set through leave-one-out cross-validation (LOOCV) and bootstrap resampling used solely for hyperparameter optimization. Specifically: (1) Training/fitting occurred within each LOOCV or bootstrap iteration, (2) the held-out samples within these resampling procedures served as validation subsets for evaluating hyperparameter configurations, and (3) the final optimized hyperparameters were then applied to the completely unseen data in the test set (25%) for unbiassed performance estimation. We have incorporated a figurative workflow to enhance the visualization of the strategy (Fig 1). Given the limited number of variants available for each protein, we selected LOOCV and bootstrap resampling rather than k-fold cross-validation to maximize data utilization for model fitting while still maintaining rigorous internal validation.

We agree that a sequence similarity-based splitting would provide stronger evidence of generalization to novel protein variants. However, to mitigate potential bias from any single random split, we repeated the entire nested cross-validation procedure in 100 unique random partitions and reported the 95% confidence intervals across all partitions to quantify performance stability. This repeated splitting approach provides a robust estimate of generalization performance. In addition, across these 100 random partitions, splits with greater sequence diversity between train and test sets inevitably occurred, partially addressing the reviewer’s concern. We acknowledge this limitation and have addressed it in the Discussion (lines 727-732).

5. I am still unclear about the strength of the association between the proposed variants and the Dis infections. In Figure 4 it seems like many of the proposed variants (particularly for OspC and RevA) are actually more prevalent in the non-Dis strains (it is unclear what the X-axis is indicating), even when the variant is found in the B-cell epitope region. This is somewhat less convincing than what is stated by the authors: „Our findings reveal that specific aa residues within these surface exposed proteins serve as critical determinants of dissemination capacity“ (lines 433-445). None of the variants found is only found in the Dis class, indeed some are even more prevalent in Non-Dis strains.

We acknowledge the reviewer comment. To clarify, in the manuscript we used “variants” to refer to different protein sequences (e.g., DbpA_1, DbpA_2), whereas the reviewer referred to “variants” as individual amino acid residues variations at specific positions (e.g., X46A, X46T). Hereafter, we refer to the later as “residues” or “residues variations” to avoid confusion.

Figures 4A, 5A, and 6A (previously Figure 4) present the predictive residue variations identified as important features from the ML models for each protein. To improve readability, we have broken the graph to 2 panels, one showing residues appeared more frequence in Dis class and one showing residues appeared more frequence in Non-dis class. The residues also sorted by their frequency in Dis or Non-dis class instead of their order in the protein sequence.

We acknowledged that many of the selected important features here found in both Dis and Non-dis class, and even more prevalent in Non-dis class, which may seem to contradict their importance in the ML models. However, this is expected because the VIP scores present overall measure of feature contribution to the model predictive performance rather than exclusive to Dis class. Additional, dissemination is multifactorial and population genetics predicts variations mixing across phenotypes. We modified the text “as critical determinants of dissemination capacity” to clarify our statement, the revised sentence now reads: “These residues are considered strongly associated with differential dissemination capacity, though their causal role in determining virulence potential requires further validation. Our findings provide molecular-level hypothesis for future investigation of Bb pathogenesis mechanisms.” (lines 656-659),

6. At the moment, the authors explore how variants in single proteins are associated with Dis capacity. The authors could explore the effect of combined variants within the same protein sequence, as well as the co-presence of different variants in the surface proteins in the same strain. Indeed, it might be interesting to know in how many of the Dis strains the proposed protein variants co-exist.

We thank the review for this insightful suggestion. We agreed that evaluating co-occurrence of different variations within the same protein sequence, and different variants/variations in different proteins in the same strains could be interesting and reveal addition patterns. We have added this in the Discussion.

Furthermore the following clarifications:

1. How many of the collection of 299 strains were initially associated with being Dis or Non-Dis?

The collected isolates were classified to 118 disseminated and 173 localized strains based on the clinical criteria. We have added the information in lines 118-121.

2. How many unique sequences were found for each protein variant? How many variants of each protein were determined to be Dis, or non-Dis? Does this deviate substantially from the strains class distribution?

The exact number of unique sequences (variants) of each protein and the number of variants classified as Dis and Non-dis groups was reported in Supplementary Table S2: OspC: 21 Dis/ 11 Non-dis; DbpA: 20 Dis/ 9 Non-dis, RevA: 26 Dis/10 Non-dis, BBK32: 19 Dis/ 14 Non-dis and P55 (11 Dis/14 Non-dis). We have added the information of the total variant for each protein in Table 1.

3. More details should be given for the selected ML methods. Why were they selected and how were they implemented?

We have added more details for the selection and implementation of ML methods in lines 223-235, 240-242.

4. More details should be given for the Variable Importance scores. How were these calculated exactly? Is each feature evaluated for its ability to discriminate between classes?

We have added more details for the Variable Importance scores in lines 282-291. We did not evaluate the individual feature ability to discriminate between classes.

5. To evaluate the tradeoff between Specificity and Sensitivity the authors might be interested in exploring the Precision-Recall curve AUC, which is perhaps more informative in class imbalance regimes.

We thank the reviewer for the suggestion. Precision, Recall, and Precision-Recall curve AUC values are now reported in the Supplementary Table S3. In the study, we applied SMOTE oversampling to the Non-dis to improve class balance. We used ROC-AUC as the primary model performance metric for hyperparameter tuning and validation on held-out test sets, but PR-AUC showed a consistent trend and did not change our conclusions. We acknowledge that SMOTE-based oversampling can generate artificial patterns may need to be validated in the future studies, however, in the current study, SMOTE helped stabilized model training given the limited and imbalance sample sizes.

6. It is unclear to me how residues are removed during pre-processing (lines 128-130). Does this mean that if the same AA is found in the same position across all sequences, then this position is not considered?

The reviewer is correct. During data preprocessing, feature columns corresponding to positions where all sequences contained identical amino acids were removed. This reduces dimensionality and excludes non-informative features that lack discriminative power. We have revised the sentence as follows: “To reduce dimensionality, feature columns that were invariant across all sequences (i.e., positions with identical aa) were removed, as these provide no discriminative power.” (lines 177-179)

Comments on code:

The provided code seems complete and is well documented. However I was unable to run the individual scripts due to various errors. For example:

When I run OHE_code.R it seems 0 sequences are processed correctly

When I run prepare Create_Input_data.R it seems like the filterVarImp function is not defined.

We thank the reviewer comment. The OHE_code.R did not process any sequence due to the unmatched identifier between the sequence.fasta and metadata.xlsx files, primarily caused by inconsistent capitalization of the protein name. We have uploaded the revised files to the data folder. The code should work now. In the Create_Input_data.R, filterVarImp is a function in caret package. We have updated the code with importing the caret package at the beginning of the script.

\# **Reviewer 2 comments**

Machine learning-driven identification of virulence determinants in Borrelia burgdorferi associated with human dissemination

Summary:

The authors performed a detailed analysis of protein sequences from infectious isolates of B. burgdorferi. Using publicly available whole-genome sequencing data, they focused on seven surface lipoproteins implicated in the pathogenesis of lyme disease caused by B. burgdorferi, and trained machine learning models able to classify protein sequences associated with disseminated infections.

Overall, the manuscript is very well-structured, detailed, and transparent about potential limitations. Nonetheless, there are some areas where its strength could be improved.

Major comments:

The main figures in the manuscript could deliver information in a better way, some of which is currently relegated to the Supporting Information. Specifically:

Figure 1 could be problematic to extract information from. The predominant message of this figure seems to be the percentage of dissemination-associated isolates per sequence for each protein. However, the individual sequences are difficult to distinguish, and this distinction does not seem to be relevant in the text. Therefore, why not reduce the information to one plot per protein (e.g., pie chart or heatmap) and combine with the more informative Figure 2? Alternatively, if the goal is to highlight the sequences exclusively associated with dissemination, they could be plotted separately, and the other sequences represented showing that most were observed in both disseminated and non-disseminated infection strains.

We thank the reviewer for the valuable suggestions. We have improved Figure 1 (now Figure 2) by reordering the sequence variants based on total sample size rather than variant name, which highlights clinically relevant pattern and facilitates information extraction. Each plot now displays the frequency (exact count) of disseminated and localized isolates associated with each sequence. This change emphasizes sequences that exhibit disproportionate or exclusive association with specific clinical outcomes. We have also rearranged and enlarged the plots to improve clarity and readability.

Similarly, Figure 3 contains details which may be unnecessary for the reader. The main conclusion here is the combined predictive performance metrics of ML models per protein. This could be simplified or broken down into subplots relevant to either the feature set, the ML algorithm, or the performance metric. Currently, the figure makes it difficult to distinguish the individual ML model performance without considerable effort. Additionally, the intensity scale should be labeled.

Information from Figure 4 is very granular. The sequences could be sorted by the ‘+’-values in each category and color-coded, to make it easier for the reader to interpret. The y-axis should also be labeled.

Figures S11-S13 provide very useful visualization for the entire subchapter 3.5 (lines 364-386 of the Results section) and demonstrate actual biological significance of ML modeling to residues predictive in protein-protein interactions. I believe they should be moved to the main text, as they constitute major conclusions inferred from the analysis.

We have reorganized the information previously distributed across the original Figure 3, 4, and Supplementary Figures S11-S13 into three consolidated figures, one for each of the 3 proteins DbpA, OspC, and RevA. The heatmap in the original Figure 3 has been disaggregated by protein to improve clarity and interpretability. The bar plots from the original Figure 4 have been reconstructed into two subpanels for each protein: The top panel displays predictive residues with higher frequency in the Dis group, while the bottom panel shows those with higher frequency in the Non-dis group. We have expanded the annotation panel for each plot, replacing the “+” symbol with a solid dot “•” and using blank space for the “-” symbol to enhance visual clarity. These modifications, combined with the promotion of Figures S11-S13 to the main text, substantially strengthen the presentation of our key biological-associated findings.

Minor comments:

p.4 line 61 “Several studies have also reported the binding of surface-exposed lipoproteins to the host extracellular matrix (ECM) such as fibronectin (24–26), glycosaminoglycans (GAGs) (27–29), and plasminogen (30–33).”

This sentence could be restructured. At first glance, it seems to mean fibronectin, GAGs, and plasminogen are the lipoproteins, and not parts of the ECM.

We thank the reviewer for pointing this out. We have rewritten the sentence for clarification. The revised sentence now reads: “Several studies have reported these surface-exposed lipoproteins bind host extracellular matrix (ECM) components, including fibronectin (24–26), glycosaminoglycans (GAGs) (27–29), as well as the plasma protein plasminogen (30–33).” (lines 80-83).

p.5 line 82 The surface-exposed proteins examined (BBK32, DbpA, OspA, OspC, P66, and RevA) were selected based on their established roles in host cell adhesion, immune evasion, and tissue invasion processes critical to bacterial dissemination.

Is there a reason why BB0406 is not mentioned here? As far as I understand, it is an outer membrane protein in Bb paralogous to BB0405 and considered less important than it for infectivity (doi: 10.1128/IAI.00803-16) but this is not discussed in the introduction.

It was a mistake that we did not include BB0406. We have added it in the revision.

p.12 line 239 “(…), we identified between 4 and 36 unique variants per protein.”

What do the authors mean by protein “variants”? This word is often seen in the context of protein isoforms but here it seems meaning unique amino acid sequences with respect to isolate. If so, it could be useful to state this explicitly, especially since the word “variants” is then used extensively.

The reviewer is correct. We use “variants” to refer to unique amino acid sequences across isolates, not protein isoforms. We have added an explicit definition in lines 127-129 (section 2.1) to avoid confusion.

p. 18 line 369, Abbreviation “TM” is never explained

“TM” stands for Template Modelling. We have provided its full spelling at the first mention (line 580).

Other points:

There are several small grammatical errors and typos throughout the manuscript which do not change the general meaning but should be addressed before publication.

p.3 line 48 “…technical challenges for sequencing, assembly, and analysis the LB genome,

ultimately limiting…”

missing “of”

p.6 line 100 “Sequences were aligned and removed signal sequences prior to feature calculations.”

signal sequences “were” removed

p. 10 line 188 “Generalization gap, defined as the difference between train and test performance (Bias

= Train – Test), quantified overfitting (positive values) or underfitting (negative values) with

comparison conducted across all stratification levels.”

missing “was”

p.13 line 259 “BB_0406 and OspA did not exhibit strong associations with clinical phenotypes, which potentially due to their low variant diversity.”

missing “is/was” or remove “which”

p. 17 line 344 “There were 57, 29, and 42 features were selected for DbpA, OspC, and

RevA, respectively.”

Unnecessary “there were”

p. 18 line 371 “suggesting that the overall folds may reasonably accurate”

missing “be”

We appreciate the reviewer's careful attention to grammatical details. We have addressed all the grammatical errors and typos identified:

p. 3, line 48 (now line 67): Fixed – added "of" before “the LB genome”.

p. 6, line 100 (now line 132): Fixed – changed "removed signal sequences prior" to "signal sequences were removed prior."

p. 10, line 188 (now line 274): Fixed – added "was" before "quantified."

p. 13, line 259 (now line 388): Fixed – removed "which" to clarify the sentence structure.

p. 17, line 344 (now line 553): Fixed – removed the redundant "There were."

p. 18, line 371 (now line 582): Fixed – added "be" to read "may be reasonably accurate."

All corrections have been incorporated into the revised manuscript.

**PCOMPBIOL-D-25-02087**

**Reviewer comments**

This manuscript presents a comprehensive machine learning (ML) framework to identify amino acid (AA) residues within well-characterized *Borrelia burgdorferi* virulence factors that predict severe human dissemination outcomes using genomic data from 299 clinical isolates. The study extends beyond the ML approach by integrating B-cell epitope prediction and host-pathogen protein-protein interaction analyses to assess the biological relevance of the identified residues. The work addresses an important public health problem, uses appropriate methodology, and provides potentially impactful results. However, several aspects require clarification and revision to improve rigor, interpretation, and presentation.

Please consider the following remarks.

1. Several terms are used ambiguously, which may cause confusion. Consistent and precise terminology should be applied for scientific accuracy. Terms “protein variants” (e.g., Line 17) or “protein sequences” (e.g., Line 122/433/Fig1 caption) or “predictive peptides” are potentially misleading, as the analyses involve translated AA sequences derived from virulence factor genes identified from WGS data rather than experimentally validated protein variants (e.g. putative proteins encoded by their respective genes or translated AA sequences). Additionally, the term “sequence” without specifying gene or AA sequences (e.g., Line 65/87/97), and phrases like “predicted predictive and epitope residues” may also be confusing.

We have clarified the terminology by explicitly defining “variants” as unique aa sequences across isolates (lines 127-129, section 2.1) and “predictive residues” as key aa residues identified by ML models to predict dissemination potential (lines 557-558, section 3.3). We have added “gene”, “protein”, or “aa” before “sequence” where ambiguity could arise (e.g., lines 109,110, 122, 124). While “sequence” alone is used throughout the manuscript, the context consistently refers to the translated aa sequences derived from the genomic data, which should be apparent from the Method workflow. We also rewrote the sentence contains the phrase “predicted predictive and epitope residues” as “ML-identified predictive residues and DiscoTope-predicted epitopes” (lines 673-674)

2. Line 22-26: Please report the number of residues identified by ML for each protein (e.g., 57 for DbpA) and specify their overlap with residues predicted by B-cell epitope analysis.

We have added the information (lines 24-25).

3. Since the primary aim of this study is to present a computational framework, the manuscript should briefly discuss prior approaches (e.g., traditional GWAS or statistic methods) and their limitations, to provide context for the advantages of an ML-based approach.

We have added a brief discussion about the limitation of the traditional methods in lines 90-95: “In contrast to traditional Genome-wide association studies (GWAS) and univariate statistical methods, which typically examine genetic variants independently under linear or additive assumptions and struggle with multiple testing corrections and identifying epistatic interaction, ML approaches can simultaneously evaluate multiple features making them adept at detecting subtle patterns in high-dimensional genomic data (37,38).”

4. The manuscript lacks sufficient details about the 299 clinical isolates (e.g., regions/countries, sampling period), making it difficult to interpret the representativeness and distribution. Please provide a brief description of the dataset, including the number of “Dis” *vs.* “Non-dis” cases, in the Methods (and abstract, if possible), even if this is a secondary dataset.

The information of the 299 isolates was incorporated in the Abstract and Methods (lines 16-17, 117-121).

The Dis and Non-dis labels were used for ML classification. The case number are different for each protein and are reported in Figure 1 and Supplementary Table S2. We moved the description for assigning ML groups from section 2.1 to section 2.3.1. (lines 188-194)

5. Line 122-123: Please provide the lengths of each gene (bp) and putative protein (AA) and indicate the remaining AAs after signal sequence removal. Also justify the rationale for removing signal sequence. If the gap character (Line 126) represents indels, please state this explicitly to avoid ambiguity and improve reproducibility.

We acknowledge the reviewer’s suggestion. The full putative aa sequences of all variants of each protein were already provided in Supplementary Table S1. Following the reviewer’s comment, we have added the sequences after removing signal sequence, the aa sequence length before and after removing signal sequence to the table. These sequences have also been deposited in the GitHub repository of the study. The provided data is adequate for reproducibility. Additionally, since gene/protein lengths vary across variants, listing all individual values in the main text would require a large table that would duplicate information already available in the supplementary materials. We therefore believe that presenting these data in Supplementary Table S1 is adequate.

We added the rationale for removing signal sequence in lines 131-133: “Since signal sequences are not functionally relevant and variations within them can introduce misalignments, signal sequences were removed prior to sequence alignment using AlignSeqs() function in DECIPHER”

In our analysis, gap characters in the aligned sequences resulted from the alignment process itself using the AlignSeqs() function in DECIPHER. We did not separately classify or validate whether these represent true indels. To improve clarity, we added “which represents potential indels as determined by sequence alignment” (lines 174-175). This clarification helps readers understand that gap characters reflect alignment-based variations rather than specifically validated indels.

6. Please list all software and tools used (e.g., Cramér’s V, sequence alignment/editing, pairwise distance calculations) and specify the parameters for DiscoTope analysis, even if default settings were applied, to ensure reproducibility.

The required information was added to the manuscripts in lines 126 & 133 (sequence alignment/editing), 135-136 (pairwise distance calculations), 158-162 (Cramér’s V), 318-319 (DiscoTope).

7. Line 259-261/406-408: Please clarify how Cramér’s V analysis handles sparse contingency tables. The weaker associations of BB_0406 and OspA may reflect factors beyond low variant diversity, such as confounding clinical/demographic variables or a less direct role in dissemination. Discussing these possibilities would provide a more nuanced interpretation. Even if variants (as collective AA compositions) show weak associations, specific AA substitutions could still correlate with clinical outcomes.

The analysis in the original manuscript did not handle sparse contingency tables. We added an analysis for contingency tables with collapsed variant categories when any cell had an expected frequency <1 or when both dissemination categories had expected frequencies < 5, ensuring that fewer than 20% sparse cells in all the contingency tables. We reported the chi-square and Fisher’s exact p-values for both the original and collapsed tables. We included the information in the Material and Methods and Results sections (lines 154-158 and 379-382).

We appreciate the reviewer’s suggestion regarding other possibilities affecting weaker associations of BB_0406 and OspA. We agree that the weaker associations of BB_0406 and OspA may reflect multiple factors. Sequence analysis revealed that both proteins are highly conserved (98.4-99.6% identity to the B31 sequence) with < 5% substitution rate. The high conservations, combined with the small number of observed variants, limits the statistical power to detect associations. As the reviewer suggested, potential cofounding by clinical or demographic variables, and a more indirect or context-dependent roles in dissemination, may also contribute. Importantly, individual variant enrichment analysis showed that OspA_10 significantly enriched in localized strained, suggesting that specific aa substitutions could correlated with clinical outcomes despite the weak overall association. We have added this discussion to the revised manuscript (lines 617-626).

8. Line 100-102: It is unclear what “distance” represents. Please report pairwise AA percent identity ranges for each putative protein relative to B31, as well as among all unique variants, to illustrate sequence diversity (e.g., percent identity with conserved counts). This would clarify whether observed variant counts result from multiple substitutions at a few positions or fewer substitutions spread across many sites (e.g., BB_0406).

The "distance" in our analysis represents the normalized Hamming distance between sequences (proportion of differing amino acids, ranging from 0 to 1), calculated using the DistanceMatrix() function in DECIPHER with method = 'overlap', includeTerminalGaps = TRUE, and correction = 'none'. We have revised the methods section to clarify it (lines 135-139). We have added detailed substitution counts, conserved counts, and percent identity values in the Supplementary Table S1 to illustrate the specific pattern of sequence variation. We have also added Table 1 which summarizes the identity ranges and substitution patterns across all seven proteins. These data clarify whether sequence diversity results from multiple substitutions at a few positions (concentrated variation) or fewer substitutions distributed across many sites (dispersed variation).

9. Line 242-251/425-429: Sample size should be considered when interpreting associations for variants with exclusive outcomes. With only a few cases, the observed exclusivity could occur by chance, reflecting under- or non-representative sampling and potentially introducing bias. Reporting the number of cases for these variants would provide important context and improve the interpretation.

We thank the reviewer for this insightful comment. We agree that sample size is critical for interpreting these associations. The number of clinical cases (Disseminated and Localized) for each variant is presented in Figure 2 and Supplementary Table S1. We have revised the text to address this concern in lines 358-364 and 645-648. We acknowledge that most of the variants with exclusive outcomes were represented by very small sample sizes with ≤ 3 isolates per variant and could arise by chance due to sampling limitations. However, these rare variants may represent true biological associations given that the isolates were collected over 30 years and may represent distinct evolutionary lineages or ecological niches.

10. Line 458-459/Fig 4: It is unclear whether the AA positions reported in the cited study (e.g., 71-86, 104-118,) directly correspond to the ML-predicted residues in this study, as alignment differences and indels can shift numbering. For example, if residues 71-86 in the cited study were based on B31, the ML-predicted residue X61K/Q may fall within that region. Using AA positions aligned to a well-established reference sequence (e.g., B31) would enable accurate comparisons across studies and improve the interpretability of residue-level analyses (e.g., X61K associated with “Dis” *vs.* X61Q with “Non-dis”).

All AA positions mentioned in cited study are based on the B31 sequence. The corresponded B31 positions for the ML-predicted residues are presented in Fig. 4B, 5B & 6B. We have added the “B31” subscript notation to clarify the reference sequence for all mentioned positions (e.g. _B31_71-86). Additionally, we have included a detailed description of the ML residue naming system in section 2.3.1 (lines 178-186), which explicitly explains how the ML positions map to B31 coordinates (e.g., X61K(B₃₁79K) indicates position 61 with a lysine residue in the ML models which corresponds to position 79 with a lysine residue in the B31 sequence).

11. The study relies on secondary data, which is a potential limitation. Are there other risk factors that could act as confounders, such as population structure of *B. burgdorferi* isolates or differences in patient demographics? If so, please discuss these possibilities and, if feasible, describe how such confounding factors were assessed or controlled for in the analyses.

We thank the reviewer for this important comment. We agree that *B. burgdorferi* population structure and patient demographic factors could act as potential confounders. The isolate dataset spans multiple geographic regions (United States and Central Europe) and a 30-year period, and lineage- or region-specific variation may therefore influence associations with dissemination phenotypes. However, because this study relied on previously published genomes and metadata, detailed demographic information and population structure variables were not available and were not explicitly controlled for. We acknowledge this as a limitation of the study and have clarified this point in the manuscript (lines 746-750). The study is intended as a proof-of-concept framework to identify candidate virulence variants and residues for future validation.

**Minor Comments:**

1. Consistent abbreviation should be maintained throughout the manuscript (e.g., “Bb” for *B. burgdorferi vs. B. burgdorferi sensu stricto*, although both refer to the same species). Also, Lyme borreliosis (LB) is the disease and should not be used as a collective term for the *Borrelia* complex (e.g., Line 34/41).

Abbreviations have been standardized throughout the manuscript: “Bb” for *B. burgdorferi sensu stricto* and “LB” for Lyme Borrelia.

2. Line 103-106: Consider providing examples of sequences identifiers (e.g., BB_0406_1 or A-1) and listing all OspC types included in the study for clarity.

We added the examples of sequences identifiers (lines 140 and 145) and mentioned all the OspC types in the study (lines 142-143).

3. Figures require formatting improvements for clarity and consistency. Specifically:
a. Ensure all axes are clearly labeled (Fig1/2/4),
b. Place shared legends for multi-panel figures in a clear, consistent location (e.g., below the panels in Fig1)
c. Consider providing categorical legends for heatmaps to clarify color coding (Fig3).
d. In Fig4, the x-axis label “binding” is used inconsistently across different protein panels; please clarify and standardize the labeling.

We have improved Figure 1 (now Figure 2) by reordering the sequence variants based on total sample size rather than variant name, which highlights clinically relevant pattern and facilitates information extraction. Each plot displays the frequency (exact count) of disseminated and localized isolates associated with each sequence. This approach emphasizes sequences that exhibit disproportionate or exclusive association with specific clinical outcomes. We have also rearranged and enlarged the plots for better visualization.

We have reorganized the information previously distributed across Figure 3, 4, and Supplementary Figures S11-S13 into three consolidated figures, one for each of the 3 proteins DbpA, OspC, and RevA. The heatmap in the original Figure 3 has been disaggregated by protein (now Figures 4A, 5A, and 6A) to improve clarity and interpretability. The bar plots from the original Figure 4 have been reconstructed into two subpanels for each protein (now Figures 4B, 5B, and 6B): The top panel displays predictive residues with higher frequency in the Dis group, while the bottom panel shows those with higher frequency in the Non-dis group. We have expanded the annotation panel for each plot, replacing the “+” symbol with a solid dot “•” and using blank space for the “-” symbol to enhance visual clarity. These modifications, combined with the promotion of Figures S11-S13 to the main text (now Figures 4C-D, 5C, and 6C), substantially strengthen the presentation of our key biological-associated findings.

4. A few references are missing (Line 35/365-366), including for NCBI BioProject PRJNA923804, the B31 reference strain, and the OspC crystal structure (PDB: 1GGQ).

We have updated all the missing references (lines 116, 124, 592).

5. Please revise minor typographical or formatting errors in Line 59/60/217/226/475/486/Fig2 caption (e.g., surface exposed, Borrelia protein variants, surface-exposed residue). BB_0406 is also missing in Line 81-82.

We thank the reviewer for carefully identifying these errors. All noted errors have been corrected, and the missing BB_0406 has been added.
