# Supplementary material for: Machine learning-driven identification of virulence determinants in Borrelia burgdorferi associated with human dissemination
Source: PLoS Comput Biol. 2026 Jun 17;22(6):e1014407. doi: 10.1371/journal.pcbi.1014407 (PMC13293517; doi:10.1371/journal.pcbi.1014407)
Supplement: Attachment — Submitted filename: Respose to Reviewers_rv2.docx [file pcbi.1014407.s024.docx]

We thank all the reviewers for their positive assessment of the revised manuscript. Below we address the additional minor comments raised by Reviewer #3.

Reviewer #3: Thank you for the revised manuscript and the detailed point-by-point responses. The revisions have adequately addressed my previous concerns. I have no major comments or additional revisions to suggest.
I do, however, have a few minor comments for consideration:
1. Lines 362-364: Please consider providing supplementary metadata for the variants (e.g., country and year) to support this.

The metadata including the country of origin and year of collection are available in the original sequencing publication (Lemieux et al., 2023) and the NCBI Project cited in the manuscript. To avoid redundancy, we did not include these as a supplementary table. We have added a statement in the Data Availability section that metadata can be retrieved via the provided NCBI accession numbers or supplementary of Lemieux et al. (2023) (lines 778-780).

2. Line 16: Replace “United State and Center Europe” with “United States and Slovenia” (typographical errors: “s” and “Center”; since samples are only from Slovenia (and the US), specifying them directly rather than using “Central Europe” improves precision).

We corrected “United State and Center Europe” to “United States and Slovenia”.

3. Figures (minor improvements):
a. Fig 3: The meaning of the annotations on the bars (n, p) appears to be missing in the figure caption.

We added the meaning of the n and p annotations in the caption (lines 394-395).

b. Fig4-6, panel B: the meaning of the dots appears to be missing in the legend/caption.

We added the explanation of the dots in the caption of Fig 4-6 (lines 433, 455, and 478).

c. Fig4-6, panel A: Adding a categorical legend instead of the current continuous scale (0–1) in the heatmaps may improve readability.

We revied the heatmap color to categorical legend in Fig4-6, as well as in the Supplementary Fig. S11.
