# Supplementary material for: Machine learning-driven identification of virulence determinants in Borrelia burgdorferi associated with human dissemination
Source: PLoS Comput Biol. 2026 Jun 17;22(6):e1014407. doi: 10.1371/journal.pcbi.1014407 (PMC13293517; doi:10.1371/journal.pcbi.1014407)
Supplement: Peer review history

# Peer review: Decision Letter 0

**DOI:** 10.1371/journal.pcbi.1014407.r001

**Authors:** James Faeder, Gaurav Sharma

8 Jan 2026

PCOMPBIOL-D-25-02087

Machine learning-driven identification of virulence determinants in *Borrelia burgdorferi*  associated with human dissemination

PLOS Computational Biology

Dear Dr. Nguyen,

Thank you for the opportunity to review your work and for submitting your manuscript to PLOS Computational Biology. We appreciate the effort and scientific rigor that went into your study. As part of the standard evaluation process, three field-specific expert reviewers have thoroughly assessed your submission. Their detailed comments, suggestions, and concerns are provided below to help you further strengthen the manuscript. After careful consideration, we feel that it has merit but does not fully meet PLOS Computational Biology's publication criteria as it currently stands. Therefore, we invite you to submit a revised version of the manuscript that addresses the points raised during the review process. Please review the enclosed feedback carefully and address each point in a systematic and comprehensive manner. We encourage you to provide clear responses and justify any changes or decisions you make during the revision process.

Please submit your revised manuscript by Mar 10 2026 11:59PM. If you will need more time than this to complete your revisions, please reply to this message or contact the journal office at ploscompbiol@plos.org. When you're ready to submit your revision, log on to https://www.editorialmanager.com/pcompbiol/ and select the 'Submissions Needing Revision' folder to locate your manuscript file.

Please include the following items when submitting your revised manuscript:

* A letter that responds to each point raised by the editor and reviewer(s). You should upload this letter as a separate file labeled 'Response to Reviewers'. This file does not need to include responses to formatting updates and technical items listed in the 'Journal Requirements' section below.

* A marked-up copy of your manuscript that highlights changes made to the original version. You should upload this as a separate file labeled 'Revised Manuscript with Track Changes'.

* An unmarked version of your revised paper without tracked changes. You should upload this as a separate file labeled 'Manuscript'.

If you would like to make changes to your financial disclosure, competing interests statement, or data availability statement, please make these updates within the submission form at the time of resubmission. Guidelines for resubmitting your figure files are available below the reviewer comments at the end of this letter

We look forward to receiving your revised manuscript.

Kind regards,

Gaurav Sharma

Academic Editor

PLOS Computational Biology

James Faeder

Section Editor

PLOS Computational Biology

**Additional Editor Comments (if provided):**

**Journal Requirements:**

If the reviewer comments include a recommendation to cite specific previously published works, please review and evaluate these publications to determine whether they are relevant and should be cited. There is no requirement to cite these works unless the editor has indicated otherwise.

[Note: HTML markup is below. Please do not edit.]

**Reviewers' comments:**

Reviewer's Responses to Questions

**Comments to the Authors:**

**Please note here if the review is uploaded as an attachment.**

Reviewer #1: Comments are uploaded as attachment.

Reviewer #2: Review PCOMPBIOL-D-25-02087

Machine learning-driven identification of virulence determinants in Borrelia burgdorferi associated with human dissemination

Summary:

The authors performed a detailed analysis of protein sequences from infectious isolates of B. burgdorferi. Using publicly available whole-genome sequencing data, they focused on seven surface lipoproteins implicated in the pathogenesis of lyme disease caused by B. burgdorferi, and trained machine learning models able to classify protein sequences associated with disseminated infections.

Overall, the manuscript is very well-structured, detailed, and transparent about potential limitations. Nonetheless, there are some areas where its strength could be improved.

Major comments:

The main figures in the manuscript could deliver information in a better way, some of which is currently relegated to the Supporting Information. Specifically:

Figure 1 could be problematic to extract information from. The predominant message of this figure seems to be the percentage of dissemination-associated isolates per sequence for each protein. However, the individual sequences are difficult to distinguish, and this distinction does not seem to be relevant in the text. Therefore, why not reduce the information to one plot per protein (e.g., pie chart or heatmap) and combine with the more informative Figure 2? Alternatively, if the goal is to highlight the sequences exclusively associated with dissemination, they could be plotted separately, and the other sequences represented showing that most were observed in both disseminated and non-disseminated infection strains.

Similarly, Figure 3 contains details which may be unnecessary for the reader. The main conclusion here is the combined predictive performance metrics of ML models per protein. This could be simplified or broken down into subplots relevant to either the feature set, the ML algorithm, or the performance metric. Currently, the figure makes it difficult to distinguish the individual ML model performance without considerable effort. Additionally, the intensity scale should be labeled.

Information from Figure 4 is very granular. The sequences could be sorted by the ‘+’-values in each category and color-coded, to make it easier for the reader to interpret. The y-axis should also be labeled.

Figures S11-S13 provide very useful visualization for the entire subchapter 3.5 (lines 364-386 of the Results section) and demonstrate actual biological significance of ML modeling to residues predictive in protein-protein interactions. I believe they should be moved to the main text, as they constitute major conclusions inferred from the analysis.

Minor comments:

p.4 line 61 “Several studies have also reported the binding of surface-exposed lipoproteins to the host extracellular matrix (ECM) such as fibronectin (24–26), glycosaminoglycans (GAGs) (27–29), and plasminogen (30–33).”

This sentence could be restructured. At first glance, it seems to mean fibronectin, GAGs, and plasminogen are the lipoproteins, and not parts of the ECM.

p.5 line 82 The surface-exposed proteins examined (BBK32, DbpA, OspA, OspC, P66, and RevA) were selected based on their established roles in host cell adhesion, immune evasion, and tissue invasion processes critical to bacterial dissemination.

Is there a reason why BB0406 is not mentioned here? As far as I understand, it is an outer membrane protein in Bb paralogous to BB0405 and considered less important than it for infectivity (doi: 10.1128/IAI.00803-16) but this is not discussed in the introduction.

p.12 line 239 “(…), we identified between 4 and 36 unique variants per protein.”

What do the authors mean by protein “variants”? This word is often seen in the context of protein isoforms but here it seems meaning unique amino acid sequences with respect to isolate. If so, it could be useful to state this explicitly, especially since the word “variants” is then used extensively.

p. 18 line 369, Abbreviation “TM” is never explained

Other points:

There are several small grammatical errors and typos throughout the manuscript which do not change the general meaning but should be addressed before publication.

p.3 line 48 “…technical challenges for sequencing, assembly, and analysis the LB genome,

ultimately limiting…”

missing “of”

p.6 line 100 “Sequences were aligned and removed signal sequences prior to feature calculations.”

signal sequences “were” removed

p. 10 line 188 “Generalization gap, defined as the difference between train and test performance (Bias

= Train – Test), quantified overfitting (positive values) or underfitting (negative values) with

comparison conducted across all stratification levels.”

missing “was”

p.13 line 259 “BB_0406 and OspA did not exhibit strong associations with clinical phenotypes, which potentially due to their low variant diversity.”

missing “is/was” or remove “which”

p. 17 line 344 “There were 57, 29, and 42 features were selected for DbpA, OspC, and

RevA, respectively.”

Unnecessary “there were”

p. 18 line 371 “suggesting that the overall folds may reasonably accurate”

missing “be”

Reviewer #3: The review is uploaded as an attachment.

**********

**Have the authors made all data and (if applicable) computational code underlying the findings in their manuscript fully available?**

The PLOS Data policy requires authors to make all data and code underlying the findings described in their manuscript fully available without restriction, with rare exception (please refer to the Data Availability Statement in the manuscript PDF file). The data and code should be provided as part of the manuscript or its supporting information, or deposited to a public repository. For example, in addition to summary statistics, the data points behind means, medians and variance measures should be available. If there are restrictions on publicly sharing data or code —e.g. participant privacy or use of data from a third party—those must be specified.

Reviewer #1: Yes

Reviewer #2: Yes

Reviewer #3: Yes

**********

PLOS authors have the option to publish the peer review history of their article (what does this mean?). If published, this will include your full peer review and any attached files.

If you choose “no”, your identity will remain anonymous but your review may still be made public.

**Do you want your identity to be public for this peer review?**  For information about this choice, including consent withdrawal, please see our Privacy Policy.

Reviewer #1: No

Reviewer #2: No

Reviewer #3: No

[NOTE: If reviewer comments were submitted as an attachment file, they will be attached to this email and accessible via the submission site. Please log into your account, locate the manuscript record, and check for the action link "View Attachments". If this link does not appear, there are no attachment files.]

**Figure resubmission:**

While revising your submission, we strongly recommend that you use PLOS’s NAAS tool (https://ngplosjournals.pagemajik.ai/artanalysis) to test your figure files. NAAS can convert your figure files to the TIFF file type and meet basic requirements (such as print size, resolution), or provide you with a report on issues that do not meet our requirements and that NAAS cannot fix.

After uploading your figures to PLOS’s NAAS tool - https://ngplosjournals.pagemajik.ai/artanalysis, NAAS will process the files provided and display the results in the "Uploaded Files" section of the page as the processing is complete. If the uploaded figures meet our requirements (or NAAS is able to fix the files to meet our requirements), the figure will be marked as "fixed" above. If NAAS is unable to fix the files, a red "failed" label will appear above. When NAAS has confirmed that the figure files meet our requirements, please download the file via the download option, and include these NAAS processed figure files when submitting your revised manuscript.

**Reproducibility:**

To enhance the reproducibility of your results, we recommend that authors of applicable studies deposit laboratory protocols in protocols.io, where a protocol can be assigned its own identifier (DOI) such that it can be cited independently in the future. Additionally, PLOS ONE offers an option to publish peer-reviewed clinical study protocols. Read more information on sharing protocols at https://plos.org/protocols?utm_medium=editorial-email&utm_source=authorletters&utm_campaign=protocols

Attachment Submitted filename: Review PCOMPBIOL-D-25-02087.pdf

Attachment Submitted filename: PCOMPBIOL-D-25-02087_Reviewer comments.pdf

Attachment Submitted filename: Reviewer 1 comments.pdf

# Author comment: Author response to Decision Letter 1

**DOI:** 10.1371/journal.pcbi.1014407.r002

5 Mar 2026

Attachment Submitted filename: Response to reviewers.docx

# Peer review: Decision Letter 1

**DOI:** 10.1371/journal.pcbi.1014407.r003

**Authors:** James Faeder, Gaurav Sharma, James Faeder, Gaurav Sharma

10 May 2026

PCOMPBIOL-D-25-02087R1

Machine learning-driven identification of virulence determinants in *Borrelia burgdorferi*  associated with human dissemination

PLOS Computational Biology

Dear Dr. Nguyen,

Thank you for submitting your manuscript to PLOS Computational Biology. Two reviewers have accepted this version; however, one has a few minor comments. Once you have rectified these minor comments, we will send a final formal acceptance. Therefore, we invite you to submit a revised version of the manuscript that addresses the points raised during the review process.

Please submit your revised manuscript by Jul 10 2026 11:59PM. If you will need more time than this to complete your revisions, please reply to this message or contact the journal office at ploscompbiol@plos.org. When you're ready to submit your revision, log on to https://www.editorialmanager.com/pcompbiol/ and select the 'Submissions Needing Revision' folder to locate your manuscript file.

Please include the following items when submitting your revised manuscript:

* A letter that responds to each point raised by the editor and reviewer(s). You should upload this letter as a separate file labeled 'Response to Reviewers'. This file does not need to include responses to formatting updates and technical items listed in the 'Journal Requirements' section below.

* A marked-up copy of your manuscript that highlights changes made to the original version. You should upload this as a separate file labeled 'Revised Manuscript with Track Changes'.

* An unmarked version of your revised paper without tracked changes. You should upload this as a separate file labeled 'Manuscript'.

If you would like to make changes to your financial disclosure, competing interests statement, or data availability statement, please make these updates within the submission form at the time of resubmission. Guidelines for resubmitting your figure files are available below the reviewer comments at the end of this letter.

As the corresponding author, your ORCID iD is verified in the submission system and will appear in the published article. PLOS supports the use of ORCID, and we encourage all coauthors to register for an ORCID iD and use it as well. Please encourage your coauthors to verify their ORCID iD within the submission system before final acceptance, as unverified ORCID iDs will not appear in the published article. Only the individual author can complete the verification step; PLOS staff cannot verify ORCID iDs on behalf of authors.

We look forward to receiving your revised manuscript.

Kind regards,

Gaurav Sharma

Academic Editor

PLOS Computational Biology

James Faeder

Section Editor

PLOS Computational Biology

**Additional Editor Comments:**

Dear Dr. Hoa Thanh Nguyen,

Two reviewers have accepted this version; however, one reviewer has a few minor comments. Once you have rectified these minor comments, we will send a final formal acceptance.

**Journal Requirements:**

If the reviewer comments include a recommendation to cite specific previously published works, please review and evaluate these publications to determine whether they are relevant and should be cited. There is no requirement to cite these works unless the editor has indicated otherwise.

1) Your manuscript's sections are not in the correct order.  Please amend to the following order: Abstract, Introduction, Results, Discussion, and Methods

**Reviewers' comments:**

Reviewer's Responses to Questions

**Comments to the Authors:**

**Please note here if the review is uploaded as an attachment.**

Reviewer #1: The authors have addressed my concerns. The manuscript is now much clearer and improved. I still have some reservations regarding the strength of the associations with each disseminating phenotype, particularly given the assignment of samples to the dis and non-dis class. However, the limitations are clearly explained.

Reviewer #2: The authors replied to all of my comments and I believe the manuscript is now much more complete and suitable for publication.

Figures 2 and 4/5/6 are now substantially improved in terms of readability and communicating information. The newly added Tables 1 and 2 also serve as a succinct comparison of the differences between the proteins and their key sequence variants, respectively.

The work seems to me methodologically sound, well-presented, and thoroughly described.

I have no further comments.

Reviewer #3: Thank you for the revised manuscript and the detailed point-by-point responses. The revisions have adequately addressed my previous concerns. I have no major comments or additional revisions to suggest.

I do, however, have a few minor comments for consideration:

1. Lines 362-364: Please consider providing supplementary metadata for the variants (e.g., country and year) to support this.

2. Line 16: Replace “United State and Center Europe” with “United States and Slovenia” (typographical errors: “s” and “Center”; since samples are only from Slovenia (and the US), specifying them directly rather than using “Central Europe” improves precision).

3. Figures (minor improvements):

a. Fig 3: The meaning of the annotations on the bars (n, p) appears to be missing in the figure caption.

b. Fig4-6, panel B: the meaning of the dots appears to be missing in the legend/caption.

c. Fig4-6, panel A: Adding a categorical legend instead of the current continuous scale (0–1) in the heatmaps may improve readability.

**********

**Have the authors made all data and (if applicable) computational code underlying the findings in their manuscript fully available?**

The PLOS Data policy requires authors to make all data and code underlying the findings described in their manuscript fully available without restriction, with rare exception (please refer to the Data Availability Statement in the manuscript PDF file). The data and code should be provided as part of the manuscript or its supporting information, or deposited to a public repository. For example, in addition to summary statistics, the data points behind means, medians and variance measures should be available. If there are restrictions on publicly sharing data or code —e.g. participant privacy or use of data from a third party—those must be specified.

Reviewer #1: Yes

Reviewer #2: Yes

Reviewer #3: Yes

**********

PLOS authors have the option to publish the peer review history of their article (what does this mean?). If published, this will include your full peer review and any attached files.

If you choose “no”, your identity will remain anonymous but your review may still be made public.

**Do you want your identity to be public for this peer review?**  For information about this choice, including consent withdrawal, please see our Privacy Policy.

Reviewer #1: No

Reviewer #2: No

Reviewer #3: No

[NOTE: If reviewer comments were submitted as an attachment file, they will be attached to this email and accessible via the submission site. Please log into your account, locate the manuscript record, and check for the action link "View Attachments". If this link does not appear, there are no attachment files.]

**Figure resubmission:**

-->While revising your submission, we strongly recommend that you use PLOS’s NAAS tool (https://ngplosjournals.pagemajik.ai/artanalysis) to test your figure files. NAAS can convert your figure files to the TIFF file type and meet basic requirements (such as print size, resolution), or provide you with a report on issues that do not meet our requirements and that NAAS cannot fix.

After uploading your figures to PLOS’s NAAS tool - https://ngplosjournals.pagemajik.ai/artanalysis, NAAS will process the files provided and display the results in the "Uploaded Files" section of the page as the processing is complete. If the uploaded figures meet our requirements (or NAAS is able to fix the files to meet our requirements), the figure will be marked as "fixed" above. If NAAS is unable to fix the files, a red "failed" label will appear above. When NAAS has confirmed that the figure files meet our requirements, please download the file via the download option, and include these NAAS processed figure files when submitting your revised manuscript.

**Reproducibility:**

To enhance the reproducibility of your results, we recommend that authors of applicable studies deposit laboratory protocols in protocols.io, where a protocol can be assigned its own identifier (DOI) such that it can be cited independently in the future. Additionally, PLOS ONE offers an option to publish peer-reviewed clinical study protocols. Read more information on sharing protocols at https://plos.org/protocols?utm_medium=editorial-email&utm_source=authorletters&utm_campaign=protocols

# Author comment: Author response to Decision Letter 2

**DOI:** 10.1371/journal.pcbi.1014407.r004

11 May 2026

Attachment Submitted filename: Respose to Reviewers_rv2.docx

# Editor report: Decision Letter 2

**DOI:** 10.1371/journal.pcbi.1014407.r005

**Authors:** James Faeder, Gaurav Sharma, James Faeder, Gaurav Sharma, James Faeder, Gaurav Sharma

4 Jun 2026

Dear Nguyen,

We are pleased to inform you that your manuscript, 'Machine learning-driven identification of virulence determinants in *Borrelia burgdorferi*  associated with human dissemination,' has been provisionally accepted for publication in PLOS Computational Biology.

Before your manuscript can be formally accepted, you will need to complete some formatting changes, which you will receive in a follow-up email. A member of our team will be in touch with a set of requests.

Please note that your manuscript will not be scheduled for publication until you have made the required changes, so a swift response is appreciated.

IMPORTANT: The editorial review process is now complete. PLOS will only permit corrections to spelling, formatting or significant scientific errors from this point onwards. Requests for major changes, or any which affect the scientific understanding of your work, will cause delays to the publication date of your manuscript.

Should you, your institution's press office or the journal office choose to press release your paper, you will automatically be opted out of early publication. We ask that you notify us now if you or your institution is planning to press release the article. All press must be co-ordinated with PLOS.

Thank you again for supporting Open Access publishing; we are looking forward to publishing your work in PLOS Computational Biology.

Best regards,

Gaurav Sharma, PhD

Academic Editor

PLOS Computational Biology

James Faeder

Section Editor

PLOS Computational Biology

***********************************************************

# Editor report: Acceptance letter

**DOI:** 10.1371/journal.pcbi.1014407.r006

**Authors:** James Faeder, Gaurav Sharma, James Faeder, Gaurav Sharma, James Faeder, Gaurav Sharma

PCOMPBIOL-D-25-02087R2

Machine learning-driven identification of virulence determinants in *Borrelia burgdorferi*  associated with human dissemination

Dear Dr Nguyen,

I am pleased to inform you that your manuscript has been formally accepted for publication in PLOS Computational Biology. Your manuscript is now with our production department and you will be notified of the publication date in due course.

The corresponding author will soon be receiving a typeset proof for review, to ensure errors have not been introduced during production. Please review the PDF proof of your manuscript carefully, as this is the last chance to correct any errors. Please note that major changes, or those which affect the scientific understanding of the work, will likely cause delays to the publication date of your manuscript.

Soon after your final files are uploaded, unless you have opted out, the early version of your manuscript will be published online. The date of the early version will be your article's publication date. The final article will be published to the same URL, and all versions of the paper will be accessible to readers.

For Research, Software, and Methods articles, you will receive an invoice from PLOS for your publication fee after your manuscript has reached the completed accept phase. If you receive an email requesting payment before acceptance or for any other service, this may be a phishing scheme. Learn how to identify phishing emails and protect your accounts at https://explore.plos.org/phishing.

Thank you again for supporting PLOS Computational Biology and open-access publishing. We are looking forward to publishing your work!

With kind regards,

Zsofia Freund

PLOS Computational Biology | Carlyle House, Carlyle Road, Cambridge CB4 3DN | United Kingdom ploscompbiol@plos.org | Phone +44 (0) 1223-442824 | ploscompbiol.org | @PLOSCompBiol
